# Mechanisms of Hemagglutinin Targeted Influenza Virus Neutralization

**DOI:** 10.1371/journal.pone.0080034

**Published:** 2013-12-11

**Authors:** Boerries Brandenburg, Wouter Koudstaal, Jaap Goudsmit, Vincent Klaren, Chan Tang, Miriam V. Bujny, Hans J. W. M. Korse, Ted Kwaks, Jason J. Otterstrom, Jarek Juraszek, Antoine M. van Oijen, Ronald Vogels, Robert H. E. Friesen

**Affiliations:** 1 Crucell Vaccine Institute, Janssen Center of Excellence for Immunoprophylaxis, Leiden, The Netherlands; 2 Centre for Synthetic Biology, Zernike Institute for Advanced Materials, Groningen, The Netherlands; 3 Harvard Biophysics Program, Harvard Medical School, Boston, Massachusetts, United States of America; University of Edinburgh, United Kingdom

## Abstract

Human monoclonal antibodies have been identified which neutralize broad spectra of influenza A or B viruses. Here, we dissect the mechanisms by which such antibodies interfere with infectivity. We distinguish four mechanisms that link the conserved hemagglutinin (HA) epitopes of broadly neutralizing antibodies to critical processes in the viral life cycle. HA-stem binding antibodies can act intracellularly by blocking fusion between the viral and endosomal membranes and extracellularly by preventing the proteolytic activation of HA. HA-head binding antibodies prevent viral attachment and release. These insights into newly identified ways by which the human immune system can interfere with influenza virus infection may aid the development of novel universal vaccines and antivirals.

## Introduction

Influenza viruses continue to be a major cause of morbidity and mortality due to shortcomings of currently available vaccines and antivirals. Despite the well-established role of neutralizing antibodies in the defense against influenza virus infection [1], [2] there is a lack of evidence on how such antibodies interfere with infection. Further understanding of their mechanisms of action, correlated to the structures involved, may guide the design of better vaccines and antivirals.

Neutralizing antibodies mainly target the hemagglutinin (HA) protein, the major envelope glycoprotein of influenza viruses. The HA protein is synthesized as a single precursor protein (HA0) and requires cleavage by host serine proteases into two disulfide-linked subunits, HA1 and HA2, for the virus to be infectious [3], [4]. The HA1 “head” subunit mediates attachment of the virus to target cells through interactions with sialic acid receptors. After endocytosis of the virus, acidification of the endosomes triggers large conformational changes in the HA2 “stem” subunit leading to fusion of the viral and endosomal membranes and release of the viral genome into the cytoplasm, allowing the infection to progress.

The vast majority of neutralizing antibodies in infected or vaccinated individuals interferes with attachment of the virus to cellular receptors by binding to exposed, highly variable loops that surround the receptor binding site. Antibodies binding to these regions are typically strain-specific and immunity following natural exposure or vaccination is mostly restricted to closely related strains. However, in the last five years, several human antibodies with remarkably broad neutralizing activity against influenza virus have been generated and characterized. Most of these broadly neutralizing antibodies (bnAbs), such as CR6261, F10, CR8020, FI6, and CR9114, were shown to bind to epitopes in the HA stem which are highly conserved among various influenza virus subtypes and have heterosubtypic neutralizing activity [5], [6], [7], [8], [9], [10]. Others, like CH65, 5J8, CR8033, and C05, bind (close) to the receptor binding site on the HA head and show broad neutralizing activity within one subtype, or neutralize selected isolates from several subtypes [10], [11], [12], [13]. Many of these bnAbs have been shown to have therapeutic efficacy in animal models [5], [7], [8], [9], [10], [12], [14], [15] and several are being developed as monoclonal antibody therapies. The broad activity of both groups of bnAbs is a result of the high level of conservation of their respective epitopes, which in turn appears to be caused by structural constraints imposed on the HA protein by the necessity to retain its key functions; receptor binding and fusion. To understand the structural basis of the broad activity, much effort has been focused on the molecular characterization of the bnAbs and their epitopes with the ultimate goal of developing a universal vaccine against influenza virus [1], [16], [17], [18]. Stem binding antibodies as well as head binding antibodies have multiple ways by which they can interfere with the viral life cycle [19], [20], [21]. Detailed knowledge on the mechanisms of action of bnAbs, as is presented here, is critical for understanding how the human immune system interferes with processes that are pivotal for influenza virus infection and spread.

## Results

### Stem-binding bnAbs are internalized by live cells in complex with viral particles, reach late endosomes, and prevent infection

**Figure 1:**
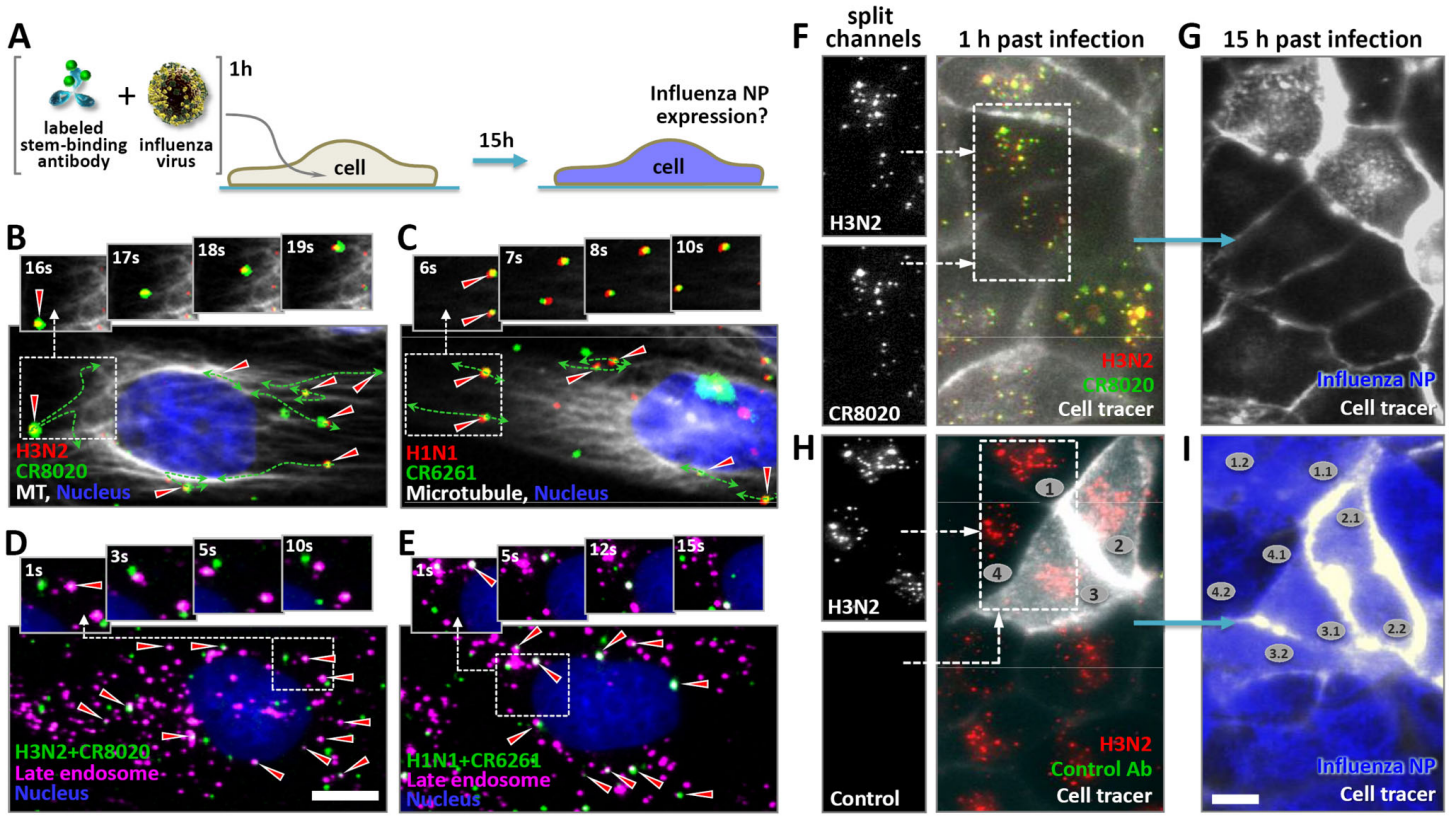
Stem-binding bnAbs are internalized into live cells in complex with viral particles, reach late endosomes, and prevent infection. (**A**) Experimental layout. Fluorescently labeled viruses and antibodies were pre-incubated and subsequently added to live cells and tracked. Whether or not cells were eventually infected was determined by staining for influenza NP after tracking individual cells for 15 hours. (**B** and **C**) Stills of movies (Movies S1 and S2) showing the joint and directed motion of R18-labeled A/Aichi/2/1968-X31 (H3N2) (red) and AF647-labeled CR8020 (green) (**B**), and R18-labeled A/Puerto Rico/8/1934 (H1N1) virus (red) and AF647-labeled CR6261 (green) (**C**), along *TubulinTracker*-stained microtubules (white) of live MDCK cells (nucleus, blue) approximately 30 minutes after addition of the pre-incubated virus-antibody mixtures. Dashed lines outline the trajectories of the virus-antibody complexes (red triangles) as seen in movies S1 and S2. (**D**) A/Aichi/2/1968-X31 (H3N2) virus was pre-incubated with AF647-labeled CR8020 (green) before addition to live MDCK cells labeled with *LysoTracker* (magenta) and imaged when virus-antibody complexes reached the perinuclear region. Arrows indicate co-localization of virus-antibody complexes with low-pH vesicles (white). (**E**) As in (**D**), except that here A/Puerto Rico/8/1934 (H1N1) virus and AF647-labeled CR6261 were used. (**F**) R18-labeled A/Aichi/2/1968-X31 (H3N2) virus (red) was incubated with AF647-labeled CR8020 (green) before addition to live MDCK cells expressing a GFP-cell tracer (grey cell outline). Virus-antibody complexes (co-localization shown in yellow, compare also split channels in the inset) were detected in live cells 30 minutes after inoculation. (**G**) To determine whether internalized virus-antibody complexes prevent infection, the fate of individual cells was assessed by tracking them over night (imaged in 30 min intervals). 15 hours post-incubation (hpi) the same cells (including their progeny) were fixed and stained for expression of influenza nuclear protein (NP, blue). (**H**) Incubation of R18-labeled A/Aichi/2/1968-X31 (H3N2) virus (red) with non-binding AF647-labeled CR6261 did not result in internalization of antibody. Only viral particles were detected in live cells 30 minutes after addition of the virus-antibody mixture and infection was not prevented, as demonstrated by the expression of NP (blue) in these same cells 15 hours later (**I**). Examples of progeny cells are indicated with numbers. Scale bars B–E equal 10 µm, F–I equal 25 µm.

Stem-binding neutralizing antibodies have been postulated to inhibit the fusion process based on their interaction with the HA2 subunit and lack of activity in hemagglutination-inhibition (HAI) assays, which specifically detect antibodies that interfere with attachment of the virus to sialic acid receptors. Indirect evidence supporting this notion comes from biochemical studies showing that such antibodies can block the conformational changes of recombinant HA required for membrane fusion [6], [8], [10], or prevent the formation of syncytia in HA-expressing cells [7], [22]. Such a mechanism of action implies that these antibodies are internalized together with the virus and reach late endosomes, but this has so far not been shown. By using fluorescence single particle tracking methods we investigated the fate of viral particles and bound antibodies during infection of live cells (Figure 1A) [23]. Movies of cells incubated with fluorescently labeled CR8020 mixed with H3N2 virus, and CR6261 mixed with H1N1 virus (CR8020 and CR6261 specifically bind Group 2 and Group 1 influenza A viruses, respectively. Table S1), reveal that stem-binding antibodies are indeed internalized in complex with the virus and transported along the microtubule cytoskeleton (Figure 1B, 1C; Movies S1, S2). The joint and directed movement of internalized viruses and bound antibodies is evident from their high degree of co-localization over consecutive frames. This behavior was exclusively observed for viruses and bound stem-binding antibodies since head-binding antibodies prevent viral internalization to begin with and no evidence for the internalization of unbound antibody could be found (Figure S1A, S1B; Movies S3, S5). Furthermore, pulse-labeling with a dye sensitive for low-pH vesicles, combined with single particle tracking in cells, demonstrated that virus-antibody complexes reach acidic late endosomes (Figure 1D, 1E). Prolonged tracking of cells that had internalized virus-antibody complexes allowed us to determine their individual fate. Following a pre-incubation with H3N2 virus, stem-binding bnAb CR8020 was observed co-localizing with viral particles inside cells at early time points (Figure 1F, Table S2). Cells were imaged every 30 minutes for 15 hours after which they were fixed and probed for the expression of influenza nucleoprotein (NP), which was used as an indicator for infection. In cells that had internalized CR8020 in complex with the virus, no NP expression was detected (Figure 1G), indicating that the bound bnAb successfully prevented infection. In contrast, a comparable number of particles led to full infection in the control experiment in which H3N2 virus had been pre-incubated with a non-binding control antibody (Figure 1H, 1I). Similar results were obtained for the inhibition of infection by H1N1 virus following pre-incubation with CR6261 (Figure S2).

### Stem-binding bnAbs prevent membrane fusion

**Figure 2:**
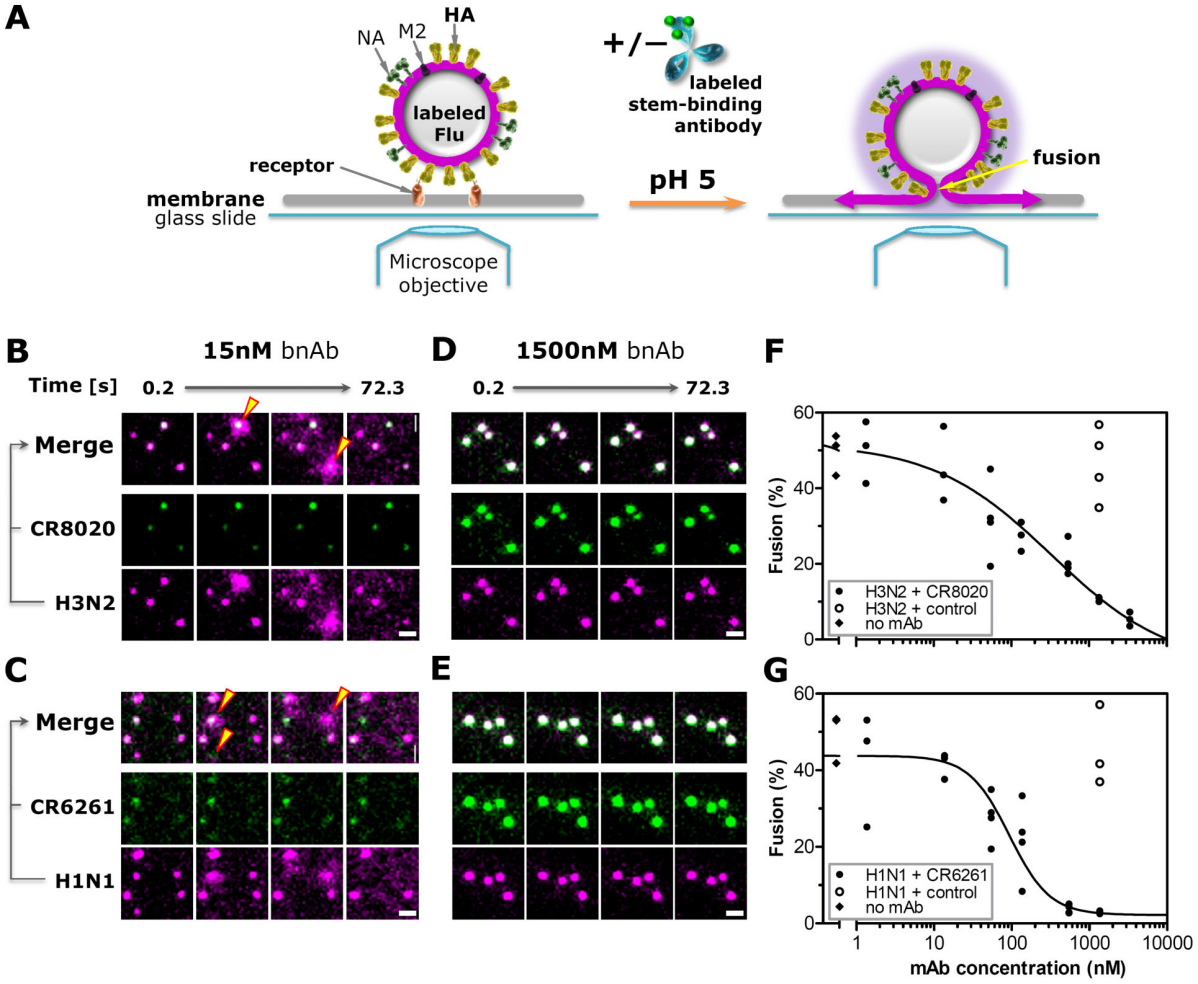
Stem-binding bnAbs prevent membrane fusion in an *in vitro* single particle fusion assay. (**A**) Assay setup in microfluidic chamber mounted on an inverted fluorescent microscope. (**B** and **D**) Stills of movies of individual R18-labeled A/Aichi/2/1968-X31 (H3N2) or (**C** and **E**) A/Puerto Rico/8/1934 (H1N1, Movie S5 and S6) virus particles (magenta) incubated with AF488-labeled bnAbs (green) and bound to sialic acid decorated proteins embedded in a supported lipid bilayer where they co-localize (white, merge). Upon lowering the pH from 7.4 to 5.0 (t = 0 seconds), viruses incubated with only 15 nM CR8020 or CR6261 undergo HA-mediated fusion with the target membrane, visualized as a rapid increase in signal due to fluorescence dequenching followed by diffusion of R18 molecules away from the fusion site (**B** and **C**, yellow triangles), whereas no fusion events occur when viruses are incubated with 1500 nM bnAbs (**D** and **E**). Scale bars equal 3 µm; illumination conditions and image contrast settings are identical in B–E. (**F** and **G**) The percentage of H3N2 and H1N1 particles undergoing fusion after the pH drop decreases with increasing concentrations of CR8020 and CR6261, respectively (black symbols). In contrast, high concentrations of bnAbs used as non-binding control antibody have no effect on the percentage of fusion (open symbols).

The finding that stem-binding bnAbs reach late endosomes in complex with the virus is congruent with the assumption that such antibodies can prevent infection by blocking fusion of the viral and endosomal membranes. To directly observe the interference of viral fusion by bnAbs, a single particle fusion assay was applied (Figure 2A). Hereto, the envelope membrane of virus particles were fluorescently labeled at a density of lipophilic dye molecules that led to fluorescence self-quenching [24], [25]. Labeled viruses were subsequently incubated with various concentrations of stem-binding bnAbs (optionally fluorescently labeled). Virus-antibody complexes were then bound to receptor proteins embedded in a target membrane and imaged. Upon lowering of the pH, HA molecules of individual viral particles incubated with a non-binding control antibody or low concentrations of bnAbs undergo conformational change and mediate membrane fusion. This event is observed as a rapid temporary increase in fluorescence signal (Figure 2B, 2C, yellow triangles; Movie S5). Increasing bnAb concentrations dramatically decrease the number of fusing virus particles (Figure 2D–G, Movie S6), demonstrating the direct inhibition of membrane fusion by stem-binding bnAbs.

### Preventing proteolytic cleavage of HA is an additional mechanism of neutralization for some stem-binding bnAbs

**Figure 3:**
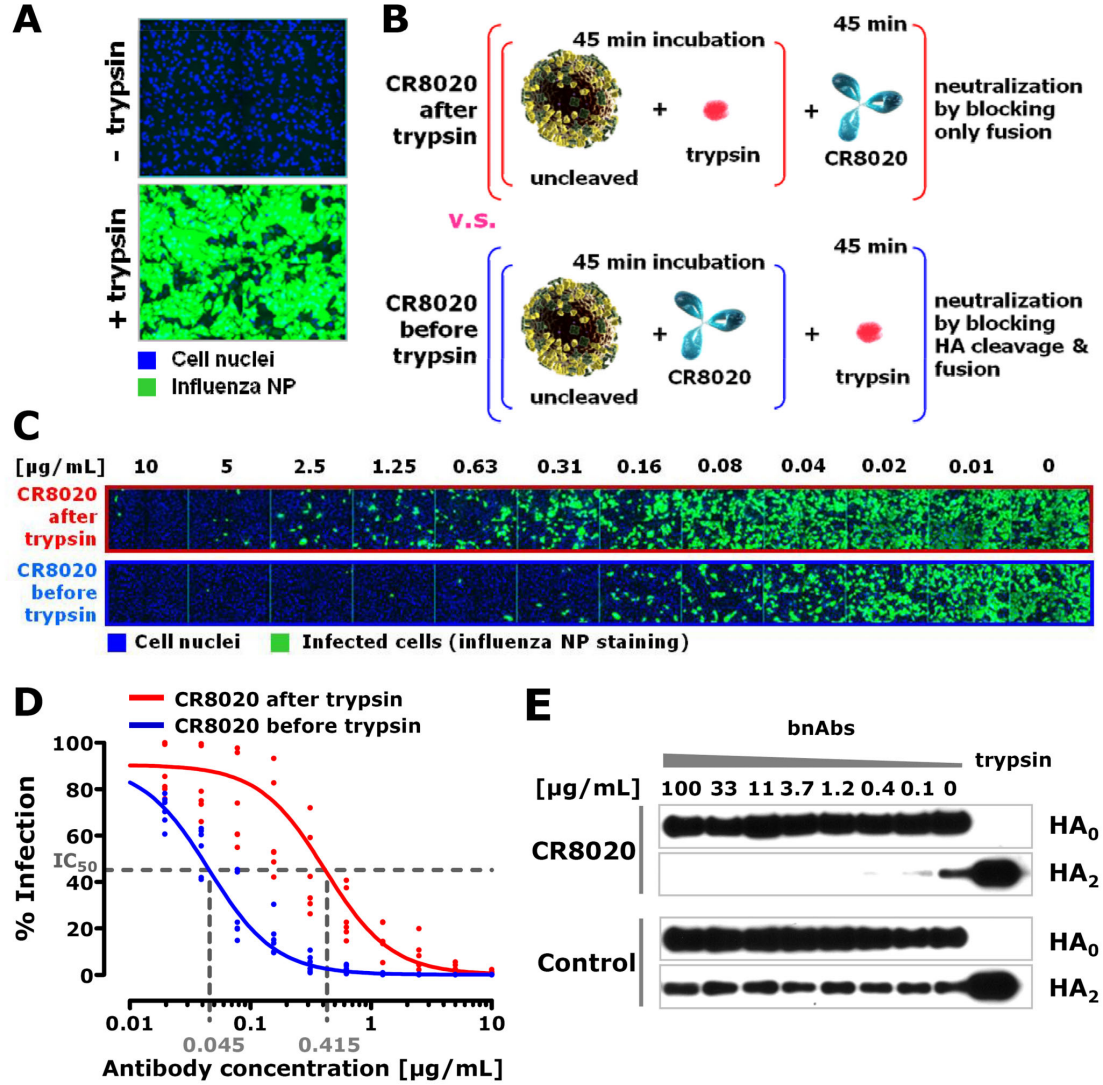
Blocking HA cleavage by CR8020 has an additive effect on virus neutralization *in vitro*. (**A**) Expression of influenza NP (green) in MDCK cells (nuclei labeled with DAPI in blue) 16 hours after inoculation with A/Wisconsin/67/2005 (H3N2) virus of which the HA was uncleaved (top) or cleaved by prior incubation with trypsin (bottom). (**B**) Experimental layout to study the additive effect of cleavage inhibition on the potency of CR8020 *in vitro*. (**C**) A/Wisconsin/67/2005 (H3N2) virus was either first incubated with trypsin and then with a serial dilution of neutralizing antibody (i.e. CR8020 after trypsin), or the virus was first incubated with serial dilutions of antibody and then treated with trypsin (i.e. CR8020 before trypsin). After 18 hours of infection, cells (nuclei stained with DAPI, blue) were stained for infection (NP expression, green). (**D**) Graph shows numerical analysis of results; normalized percentage of infection versus antibody concentration was used to compare the IC_50_ values for each condition. Change in IC_50_ is 9.2-fold (95% C.I. 6.8–12.3). (**E**) Calu-3 cells (polarized human lung epithelia) were infected with cleaved A/Wisconsin/67/2005 (H3N2). Virus was washed away after 2 hours, and cells were incubated with test and control antibody for 18 hours in the absence of trypsin. Newly produced viral particles released into the culture supernatant were harvested and the HA cleavage status was analyzed by Western blot (using rabbit polyclonal anti-HA serum). The presence of the HA2 band is indicative for cleavage (the HA1 band is not efficiently stained by the polyclonal serum).

The inhibition of the fusion between the virus and the endosome is a mechanism shared by all neutralizing stem binding bnAbs described to date. Inhibiting the cleavage of HA0 into HA1 and HA2 fragments removes the fusogenic potential of HA and is a second mechanism adding to the potency of some of the stem binding Abs. Stem-binding bnAbs CR8020 and FI6 recognize epitopes which partially overlap with the fusion peptide and bind close to the cleavage site of HA [8], [9]. Both have been reported to not only inhibit the conformational change of HA, but to also prevent trypsin from cleaving the extracellular domain of purified HA *in vitro* [8], [9]. To test the contribution of inhibiting HA cleavage on the potency of CR8020, we generated a batch of H3N2 virus of which the HA proteins were uncleaved by harvesting the virus after a single round of infection in the absence of trypsin. As expected, such ‘uncleaved’ virus was only infectious on MDCK cells after treatment with trypsin (Figure 3A). Next, we compared the potency of CR8020 against this virus treated with trypsin either before, or after addition of the antibody (Figure 3B). When CR8020 was added before cleavage, a nine-fold increase in potency was found compared to when antibody was added to previously cleaved virus (Figure 3C, 3D). This difference shows that the *in vitro* neutralizing potency of CR8020 is based on prevention of both fusion and cleavage. However, although porcine trypsin is widely used to render influenza viruses infectious in cell culture, in human lungs cleavage is thought to be mediated by membrane-bound proteases such as TMPRSS-2, and -4 and Human Airway Trypsin [26] and potentially also by secreted proteases like tryptase Clara, miniplasmin, and ectopic anionic trypsin [3]. Human lung derived Calu-3 cells form polarized epithelia and express TMPRSS-2 and -4 [27]. These cells allow the propagation of influenza virus in the absence of trypsin, indicating that cellular serine proteases are capable of mediating cleavage of progeny virus (Figure S3A). Interestingly, uncleaved virus is not infectious when added to Calu-3 cells, suggesting that cell-associated proteases are unable to cleave the HA of ‘incoming’ virus particles (Figure S3B). In order to compare the cleavage status of the HA on viral particles produced in the presence and absence of CR8020, we infected Calu-3 cells with H3N2 virus and added the antibody two hours later. In this way we prevented interference of the antibody with the initial infection, but allowed it to bind immediately to newly expressed HA molecules on the cell surface. Virus particles were harvested from the supernatant 20 hours post infection and analyzed by Western blot. Whereas in the presence of a non-binding control antibody (CR6261) a portion of the HA molecules on viral particles were cleaved, as indicated by the presence of the HA2 band, CR8020 efficiently blocked HA cleavage at a concentration as low as 0.4 µg/mL (Figure 3E). Interestingly, not all HA molecules incorporated in viral particles need to be cleaved to allow spread of infection in Calu-3 cells, as apparent from the observation that virus spreading in these cells in the absence of trypsin (Figure S3A) contains both cleaved and uncleaved HA (Figure 3E). Nevertheless, in the presence of sufficient amounts of CR8020, newly budded viral particles contain only uncleaved HA molecules, rendering them non-infectious. This shows, in a physiological situation, that CR8020 inhibits cleavage and spread of influenza virus and that a single type of antibody can act intra-cellularly (fusion inhibition) as well as extra-cellularly (cleavage inhibition).

### HA head-binding antibodies not only block attachment, but also viral egress

**Figure 4:**
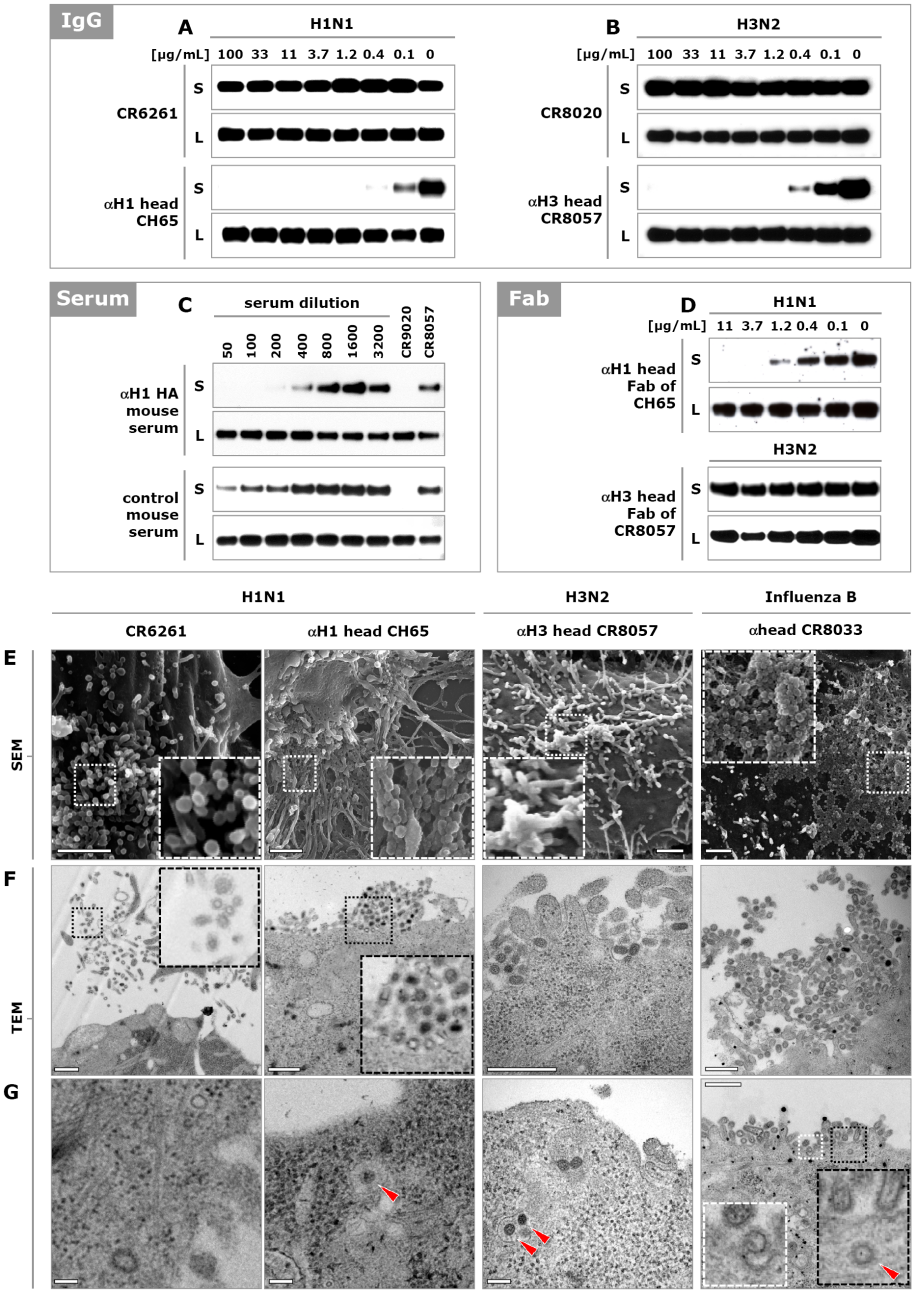
HA head binding antibodies inhibit influenza virus egress. (**A**) Calu-3 cells were infected with A/Puerto Rico/8/1934 (H1N1) and 3 hours later stem-binding antibody CR6261 or head-binding antibody CH65 was added. Twenty hours later, the amounts of HA present in the cell supernatant (S) and lysate (L) were analyzed by Western blot (HA0 band shown). (**B**) As in (A) except that cells were infected with A/Wisconsin/67/2005 (H3N2) virus and stem- and head-binding antibodies CR8020 and CR8057, respectively, were used. (**C**) Naïve mice were immunized and boosted twice with DNA encoding the HA of influenza A/Brisbane/59/2007 (H1N1) virus. Serum was collected and added to MDCK cells 3 hours after infection with the same virus. The amount of newly produced particles in culture supernatants and cell lysates were analyzed 20 hours later by Western blot (HA0 band shown). As positive and negative controls 1 µg/mL of CR9020 and CR8057 were included, respectively. (**D**) Fab fragments of head-binding antibodies CH65 and CR8057 were added 3 hours after infection of MDCK cells with A/Puerto Rico/8/1934 (H1N1) and A/Wisconsin/67/2005 (H3N2) virus, respectively, and 20 hours later the amounts of HA present in the supernatant were analyzed as above. (**E**) SEM images of the surface of MDCK cells infected with influenza A/New Caledonia/20/1999 (H1N1), A/Wisconsin/67/2005 (H3N2), or influenza B/Florida/04/2006 virus and subsequently incubated (from 3 hours post infection) with CR6261 (50 µg/mL, 333 nM), CH65 (10 µg/mL, 67 nM), CR8057 (0.5 µg/mL, 3 nM) or CR8033 (2.5 µg/mL, 17 nM) respectively. Representative images of three independent experiments are shown. Scale bar 1 µm. (**F** and **G**) As in (E) except TEM images of ultrathin sectioned MDCK cell (re-internalized particles indicated with red triangles). Scale bar in (F) 500 nm and in (G) 100 nm.

Head-binding neutralizing antibodies are well-documented to prevent viral attachment to the receptor. However, we have recently described two bnAbs, CR8033 and CR8071, which bind to the globular head of influenza B HA and are able to inhibit viral egress [10]. Whereas CR8033 also interferes with attachment of the virus to its cellular receptor, egress inhibition appears to be the only neutralization mechanism of CR8071. Since both head- and stem-binding antibodies can bind to HA on the surface of infected cells (Figure S1C), we hypothesized that egress inhibition is a more common mechanism of action for antibodies directed against HA of influenza A and B. To test this, cells were infected and three hours later, various stem- and head-binding antibodies were added (see Table S1). Delaying the addition of antibodies ensured unhindered initial infection and allowed assessment of the effect on egress only. Twenty hours after infection, the amounts of newly produced viral particles present in the supernatants and cell lysates were analyzed. Since the presence of the neutralizing antibodies would interfere with assays assessing virus titers (e.g. TCID_50_), we used Western blot analysis to determine the amount of virus. As observed with influenza B specific antibodies CR8033 and CR8071, the presence of head-binding antibodies against influenza A viruses of the H1N1 (CR9020, CH65 and 2D1) and H3N2 (CR8057) subtypes led to a significant reduction in the amount of viral particles released into the supernatant (shown by the absence of HA), while the production and accumulation of HA in the cell was not affected (Figure 4A, 4B and S5A). In contrast, the presence of HA stem-binders (CR6261 and CR8020) had no effect on the amount of viral particles released into the supernatant. Thus, egress inhibition appears to be a common mechanism of antibodies directed against the head region of HA of both influenza A and B viruses. Since head-binding antibodies are dominant in the response to infection or vaccination, we were interested to see whether polyclonal serum (besides the well-documented inhibition of receptor interaction) could also inhibit viral egress. Indeed, addition of HA-specific polyclonal mouse serum to infected cells caused a concentration dependent reduction of viral particles in the supernatant, without affecting the accumulation of HA in the cell (Figure 4C).

Confirmation that head-binding antibodies inhibit egress comes from Scanning EM (SEM) images showing that whereas separate budding particles are present at the surface of infected cells in the presence of stem-binding antibody CR6261, large aggregates of particles are visible in the presence of each of the head-binding antibodies (Figure 4E and S4, S5C). Transmission EM (TEM) images further reveal that the aggregated virions resemble fully formed free virus particles, with an electron dense core due to the vRNPs and spike proteins on the surface (Figure 4F, 4G and S4, S5D, S5E). Moreover, completely formed viral particles surrounded by an endosomal membrane were detected in the cytoplasm near the surface, suggesting that un-budded particles can be re-internalized (red triangles in Figure 4G and S5E). In all these aspects, the phenotype is similar to what is seen with the antiviral drug zanamivir, which inhibits egress by blocking the enzymatic activity of the neuraminidase (NA) protein (Figure S5C–E).

We hypothesized that HA head-binding antibodies inhibit egress by cross-linking of newly formed virions to each other and to HA on the cell membrane. In line with this hypothesis, the presence of the monovalent Fab fragments of CR8057, CR8033 and CR8071 had no effect on the amount of HA in the supernatant of cells infected with H3N2 and influenza B virus, respectively (Figure 4D and Figure S5B). Interestingly however, the Fab fragment of CH65 did result in a reduction of HA in the supernatant of cells infected with H1N1 virus, similar to the IgG molecule. Considering the phenotypic resemblance with zanamivir, one may speculate that CH65 prevents NA from performing its function through steric hindrance, rather than through cross-linking newly formed virions. However, it is also possible that all these antibodies inhibit egress in the same way (be it through hindrance of NA or otherwise), but that differences in affinity, or the orientation in which they bind to HA determines whether the Fab alone or the larger IgG molecule is required. Either way, our results show that many, if not all, head-binding neutralizing antibodies, next to preventing attachment to the receptor, also inhibit egress.

## Discussion

**Figure 5:**
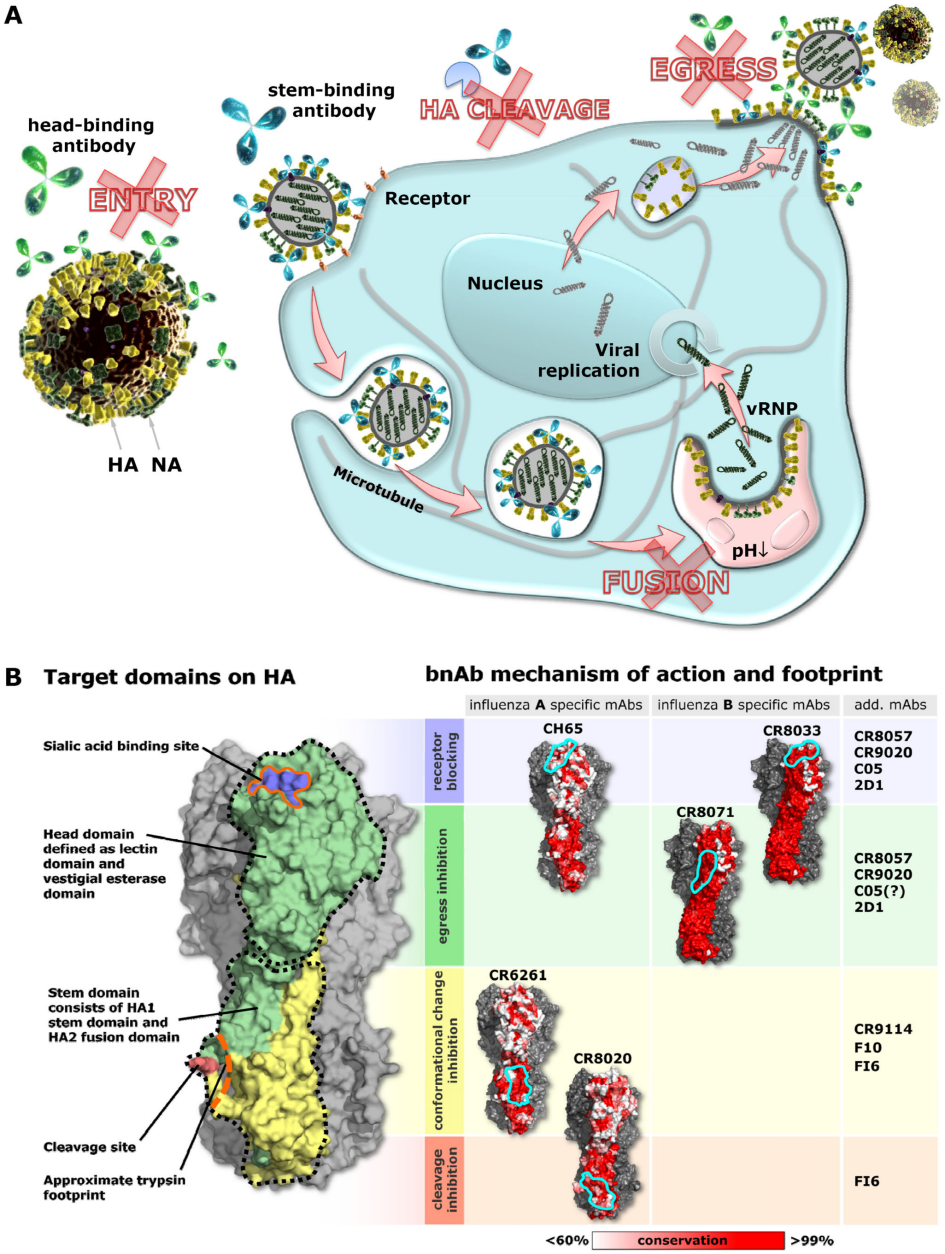
Mechanisms of action of bnAbs map to conserved regions on HA and thereby reveal conserved vulnerabilities of influenza virus. (**A**) Influenza virus life cycle highlighting the four distinct mechanism of actions of HA head-binding (green) and stem-binding (blue) bnAb. (Panel **B**, left) X-ray structure of an uncleaved H3 trimer (A/Hong Kong/1/68 PDB 1HA0) in a space filling representation. For clarity, only one monomer of the trimer is colored (HA1 green, HA2, yellow). The head region, comprising lectin and vestigial esterase domains, and the stem region, containing the fusion machinery, are indicated with dotted black lines. The receptor binding site is plotted in blue and the cleavage site in pink. The regions around these sites (solid orange lines) are the footprints of sialic acid and trypsin, respectively. To roughly estimate the trypsin footprint, a trypsin structure (PDB 1YF4) was docked on the HA cleavage site such that the cleaved HA arginine overlapped with the bound arginine from 1YF4. HA amino-acids within 5A from trypsin were then taken as an approximation of the footprint. (Panel **B**, right) Footprints, indicated by solid cyan lines, of the bnAbs studied here superimposed on HA: CH65 and CR6261 footprints are plotted on HA from A/South Carolina/1/1918 (PDB ID 3GBN), and the CR8020 footprint on A/Hong Kong/1/1968 HA (PDB ID 3SDY). For the flu B antibodies, the B/Brisbane/60/2008 structure (PDB ID 4FQM) is used. Each of the HA structures has been colored with amino-acid conservation index, corresponding to their respective virus groups: H1 – group1, H3 – group 2 and B – entire influenza B. Conservation was calculated based on the NCBI flu database set as of December 2011, assuming a number of conservative substitutions [8]. Red color corresponds to more than 99% conservation, white to less than 60% conservation. Additional human antibodies of which the epitopes and/or mechanism(s) of action are known are indicated on the far right.

The *in vivo* activity of antiviral antibodies is thought to be a combination of direct mechanisms of action (e.g. neutralization) and indirect mechanisms of action, generally mediated by immune cells (e.g. NK cells) or complement factors interacting with the Fc-tail of the bound antibodies and inducing cell toxicity [28], [29]. In this study, we focused on the direct mechanisms of action of bnAbs as a consequence of their binding to different epitopes on HA. By using live cell imaging and infectious viruses we distinguish four physiologically relevant mechanisms by which anti-HA antibodies can interfere with the pivotal functions of HA and neutralize the virus: inhibition of receptor binding, inhibition of membrane fusion, inhibition of HA0 cleavage and inhibition of egress. These mechanisms, being so diverse and tailored to different stages in the life-cycle of the influenza virus (Figure 5A), are not readily captured in a single assay format. Consequently, when assessing the potency of a particular antibody, antiviral, or a (universal) vaccine, it will be necessary to use various assays. Indeed, the use of HAI and standard microneutralization assays is one of the reasons why the existence of bnAbs has long gone undetected [30]. Likewise, some of the head-binding antibodies described previously may in addition to preventing attachment also inhibition egress [11], [13], [31], [32], [33]. Because the epitopes of the bnAbs studied here (and several others) are known, we can link their mechanisms of action to specific regions on the HA molecule (Figure 5B). Although this link is not absolute in the sense that only antibodies binding to these regions exert these mechanisms [34], the bnAbs show us highly conserved sites where interference with crucial processes involving HA is possible (Figure 5A, 5B). This information may be exploited to design broad-spectrum anti-influenza virus molecules since the broad reactivity of these antibodies means that antivirals mimicking their mechanisms of action will be broadly active, provided that they bind to the same highly conserved regions on HA.

## Materials and Methods

### Cell Culture

Suspension PER.C6® (sPER.C6®) cells [35], [36] were cultured in Adenovirus Expression Medium (AEM, Invitrogen) supplemented with 4 mM L-glutamine and passaged twice weekly. Cells were cultured at 37°C, 10% CO_2_ in a shaking incubator. The canine kidney cell line MDCK (ATCC, CCL-34) was cultured in Dulbecco's Modified Eagle Medium (DMEM) supplemented with 10% fetal bovine serum and 2 mM L-Glutamine and passaged twice weekly. The lung adenocarcinoma cell line Calu-3 (ATCC, HTB-55) was cultured in the same medium supplemented with Non Essential Amino Acids and passaged once a week. Cells were cultured at 37°C, 10% CO_2_. All culture reagents were purchased from Invitrogen (Carlsbad).

### Viruses

Purified wild type influenza viruses A/Puerto Rico/8/1934 (H1N1) and A/Aichi/1968-X31 (6∶2 reassortant of A/Puerto Rico/8/1934 with the HA and NA segments of A/Aichi/1968 (H3N2)) propagated in eggs were purchased from Charles River Laboratories and used for live cell imaging. Stock samples were certified to contain 2 mg of protein per mL and stored at −80°C. A/New Caledonia/20/1999 (H1N1), A/NYMC/X-181 (6∶2 reassortant of A/Puerto Rico/8/1934 with the HA and NA segments of A/California/07/2009 (H1N1)), A/Puerto Rico/8/1934 (H1N1), A/New Caledonia/20/1999 (H1N1), A/Brisbane/59/2007 (H1N1), A/Wisconsin/67/2005 (H3N2), A/Aichi/2/1968-X31 (H3N2), A/NYMC/X-161B (A/Puerto Rico/8/1934 with the HA and NA segments of A/Wisconsin/67/2005 (H3N2)), and B/Florida/04/2006 were grown by infecting sPER.C6® cells with virus at MOI 1×10^−4^ in infection medium (AEM and VP-FSM (2∶1), supplemented with 2.6 mM L-glutamine and 3 µg/mL trypsin (all reagents from Invitrogen)). After 72 h of incubation, virus containing cell culture supernatant was harvested by centrifugation at 4000 g for 10 min. Virus aliquots were stored at −80°C. For colocalisation and entry studies in live cells, A/New Caledonia/20/1999 and A/NYMC/X-161B were purified by ultracentrifugation at 27,000 rpm for 2 h at 4°C through a 25% sucrose cushion. The virus pellet was resuspended in NTE buffer (150 mM NaCl, 10 mM Tris, 1 mM EDTA), pH 7.4 overnight at 4°C before aliquotting and storage at −80°C.

Uncleaved viruses were produced by infecting sPER.C6® cells with cleaved virus at MOI 2 for 2 h in infection medium without trypsin. Cells were subsequently washed extensively with 10% FBS in PBS and incubated in infection medium in the absence of trypsin. Virus supernatant was harvested by centrifugation at 4000 g for 10 min. All incubations were done at 35°C, 10% CO_2_, on a shaking platform. Uncleaved status of HA was confirmed by Western blotting after probing with H1-HA or H3-HA specific polyclonal serum and infection assays to confirm the absence of infection without prior treatment with 5 µg/mL trypsin for 30 min at 37°C.

All viruses were specifically titrated to reach >90% infection in each of the experimental conditions. Controls confirmed the successful infection in every experiment.

### Antibodies (IgG expression, Fabs and polyclonal sera)

Fully human IgG1 antibodies CR6261, CR8020, CR8033, CR8057, CR9020, CR11054, and CR11055 were constructed and expressed as described previously (Ekiert et al., 2011). Fab fragments were obtained by IdeS digestion of antibodies, followed by purification via protein G (GE Healthcare), cation exchange (MonoS, GE Healthcare), and gel filtration (Superdex200, GE Healthcare). All antibodies and Fab fragments were more than 97% pure and monomeric. Influenza A nucleoprotein (NP) specific monoclonal mouse antibody was obtained from Abbiotec (clone 5D8) and for influenza B NP from Santa Cruz (sc-52027). Goat F(ab′)_2_ anti-mouse- or anti-human Alexa Fluor conjugated secondary antibodies (Invitrogen) were used for fluorescent imaging at 2 µg/mL. HA specific rabbit polyclonal serum for Immunoblot analysis was obtained from Protein Sciences. Secondary HRP-coupled anti-rabbit F(ab′)2-fragment were purchased from Jackson Immuno Research Laboratories (111-036-047). Polyclonal sheep sera directed against B/Florida/4/2006 (07/356, sheep 478 and 479) were obtained from the NIBSC and derived from sheep immunized with the respective purified HA. HA-specific polyclonal serum was derived from mice immunized intramuscularly three times at a 3-week interval with 50 µg plasmid DNA encoding full-length A/Brisbane/59/07 HA, codon-optimized for mammalian expression, mixed with 50 µg plasmid DNA encoding murine Granulocyte Macrophage-Colony Stimulating Factor (GM-CSF).

In all experiments antibodies were either used at a range of concentrations or at sufficiently high concentration to neutralize the virus under the given experimental settings. This was confirmed by neutralization controls in every experiment. Imaging also confirmed that the used antibody concentrations were sufficient to binding nearly 100% of viral particles including infectious and potentially non-infectious particles (Figure 1, Figure S1, Table S2).

### Virus labeling

Purified and concentrated viruses were diluted in HNE buffer (5 mM Hepes, 140 mM NaCl, 0.2 mM EDTA, pH 7.4) for labeling. The lipophilic fluorescent dye, Octadecyl Rhodamine B chloride (R18, Molecular Probes) dissolved in DMSO or DMSO alone as a mock labeled control was added to the samples to a final dye concentration of 1–2 µM and 0.4–0.5% DMSO. The samples were mixed for 2–3 h at room temperature, protected from light. Unincorporated dye was removed by passing the virus-dye solution through a PD-10 desalting column (GE Healthcare). Fractions containing labeled virus were pooled and labeling verified by fluorescence microscopy.

To confirm that labeling did not affect the infectivity of viruses, labeled- and mock-labeled virus samples were compared in imaged based infection assays (Figure S6). Only batches of labeled virus showing less than 2 fold differences in titer were used.

### Antibody labeling

For imaging studies, HA-specific monoclonal antibodies were fluorescently labeled according to manufacturer's guidance with the amine reactive dyes (Molecular Probes) Alexa Fluor 488 (AF488) or Alexa Fluor 647 (AF647). Briefly, dye dissolved in DMSO was added to antibodies diluted in sodium bicarbonate buffer to a basic pH. For each antibody different dye concentrations where tested to avoid over-labeling. Contents were mixed and incubated for ∼2 h protected from light. Free dye was removed from the sample by desalting and buffer exchange using PD-10 sephadex G-25 columns (GE Healthcare). Antibodies were labeled with 3–8 dyes per IgG molecule.

The biological activity of all labeled antibodies was confirmed and compared to unlabeled antibodies in viral neutralization assays before they were used in imaging experiments. Only batches of labeled antibodies showing less than 2 fold differences in titer where used.

### Virus Neutralization Assay (VNA)

MDCK cells were seeded on the day of experiment at 40,000 cells/well into 96-well flat bottom plates. Antibodies were serially diluted, mixed with an equal volume of viral inoculum and incubated for 2 h at 37°C in medium (DMEM supplemented with 2 mM L-glutamine and 3 µg/mL trypsin-EDTA). The mixture (∼100 TCID_50_/well) was then added to confluent MDCK monolayers in quadruplicate. Cells were cultured for 72 h before supernatant was added to an equal volume of 1% Turkey red blood cells and incubated for 1 h at room temperature in a 96-well V-bottom plate. The absence of hemagglutination was defined as protection. Titers were determined using the Spearman-Kärber formula.

### Hemagglutination inhibition (HI) Assay

Virus was diluted to 8 HA units/50 µL and 25 µL was combined in quadruplicate wells with an equal volume of antibody serially diluted in PBS. Plates were incubated for 1 h at 37°C in 96-well V-bottom plates. 50 µL of 1% Turkey red blood cells was then added to each well and incubated for 1 h at room temperature. Button formation was scored as evidence of hemagglutination inhibition. Titers were determined using the Spearman-Kärber formula.

### Imaging

All experiments were performed using black flat bottom 96-well imaging plates (BD Falcon) which were sealed with oxygen permeable film (Sigma Aldrich) before imaging. Images were taken after laser-based auto-focusing using a Pathway 855 high content imager (Becton Dickinson) equipped with different objectives (Olympus: 4X 0.16 NA, 20X 0.75 NA, and 40X 0.90 NA). Movies were taken with the 40X objective at4 frames/s while alternating between two channels over the duration of 3–5 min. For the overnight tracking of cells images were automatically taken at pre-defined positions over the duration of ∼15 h at ∼30 min intervals. Confirming infection (NP expression) after fixation and staining of the cells was carried out at the same pre-defined positions with the 40X objective, and also throughout the well with a 20X objective to determine the percentage of infected cells. Images of individual channels where overlaid and movies were compiled using ImageJ software [37]. Due to limited recording speed and alternating channels the registration of fast moving particles is not perfectly synchronized leading sometimes to the artificial separation of virus and antibody signal in adjacent movie frames. To determine the percentage of infected cells, image channels (e.g. cell nucleus and cytoplasm) were analyzed and segmented using Attovision software (Becton Dickinson) followed by the IC_50_ value calculation by SPSS software (IBM) and graphs plotted using GraphPad Prism software.

### Imaged based infection assay

Cells were infected with an MOI of 3 for at least 15 h and then rinsed twice with PBS followed by fixation with 80% ice cold acetone for 10 min. After removing the acetone and drying the wells the plates were washed 3 times with 300 µL per well wash buffer (PBS, 0.05% Tween-20) then incubated for 1 h with mouse anti-influenza NP antibody (1 µg/mL) in antibody dilution buffer (1% BSA, 0.1% Tween-20 in PBS) at room temperature. After washing three times with 300 µL wash buffer the wells were incubated with 2 µg/mL goat-anti mouse AF488 labeled secondary antibody and 1 µg/mL 4′,6-diamidino-2-phenylindole (DAPI) for 1 h. After three wash steps buffer was replaced with 100 µL PBS containing 0.25 mM Sodium Azide, plates sealed, and imaged.

### HA-specific staining of particles and infected cells

All staining steps described below were performed for 1 h at room temperature in the dark. ***Viral particles***: R18-labelled A/New Caledonia/20/99 (H1N1), A/Puerto Rico/8/34 (H1N1), A/NYMC/X-161B (H3N2) or A/Aichi/68-X31 (H3N2) virus was diluted in CO_2_-independent medium (Invitrogen) supplemented with 2 mM L-glutamine and spotted onto glass bottom 96-well imaging plates for 30 min at 37°C before washing with PBS and staining with anti-HA specific antibodies at 5 µg/mL in 1% BSA/PBS, followed by detection with 2 µg/mL goat anti-human-Alexa Fluor 647 secondary antibody. Wells were washed four times with medium before replacing with CO_2_-independent (phenol red free) medium (Invitrogen) supplemented with 2 mM L-glutamine for imaging.

***Infected cells***: MDCK cells were infected overnight with virus serially diluted in DMEM supplemented with 2 mM L-glutamine before fixing with either 3% paraformaldehyde (PFA) in PBS or ice cold 80% acetone for 10 min. Staining was carried out as mentioned for viral particles under both permeabilizing (acetone) and non-permeabilizing (PFA) conditions. Under permeabilizing conditions, cells were also stained for influenza NP to confirm the presence of viral infection. Nuclei were counterstained with 0.1 µg/mL DAPI.

### Virus entry inhibition

An immunofluorescence entry assay was designed to assess the ability of HA head-binding antibodies to prevent viral internalization into cells. R18-labelled H1N1 or H3N2 (MOI 3) was pre-incubated with Alexa-Fluor 647 labeled HA-specific antibodies to a final concentration of 30 µg/mL (200 nM) for 1 h at 37°C before being added to MDCK cells seeded in 96-well black-sided imaging plates (Becton Dickinson). MDCK cells stably expressing a GFP cell marker (OriGene, Rockville, USA) were incubated with virus for 15 min at 37°C followed by treatment of the cells with 0.05 U/well neuraminidase (Sigma) for 5 min at 37°C to remove non-internalized viruses. Cells were washed twice with PBS before imaging live in CO_2_-independent medium supplemented with 2 mM L-glutamine.

### Virus internalization

An immunofluorescence internalization assay was designed to assess the ability of HA stem-binding antibodies to be internalized into cells in complex with infectious virus particles. A pre-determined amount of R18-labelled H1N1 or H3N2 virus giving rise to 90–100% infection under the following experimental conditions was pre-incubated with Alexa-Fluor 647 labeled HA-specific bnAbs to a final concentration of 30 µg/mL (200 nM) for 1 h at 37°C. MDCK cells were treated for 5 min at 37°C with the cell permeant nuclear counterstain Hoechst 33342 (10 µg/mL, Invitrogen), followed by treatment with 3 µM tubulin tracker green reagent (Molecular Probes) for 30 min at 37°C to stain the microtubules. Cells were then incubated for 15 min at 37°C with the prepared virus-antibody mixture, followed by treatment with 0.05 U/well neuraminidase (Sigma Aldrich) for 5 min at 37°C. All reagents were diluted in CO_2_-independent medium supplemented with 2 mM L-glutamine. Cells were washed four times in medium before imaging live in CO_2_-independent medium supplemented with 2 mM L-glutamine and the glucose oxidase/catalase oxygen scavenging system (GODCAT, 1% glucose, 0.5 mg/mL glucose oxidase, 40 µg/mL catalase; all reagents from Sigma) to prevent photobleaching [38]. To avoid a decrease in cell viability and viral replication the exposure with light and oxygen scavenging system was limited to two hours and the medium then replaced. Movies were captured at manually selected positions with a 40X 0.90 NA objective.

### Virus colocalization

Mock-labelled H1N1 or H3N2 virus was pre-incubated with Alexa-Fluor 647 labeled anti-HA bnAbs to a final concentration of 30 µg/mL (200 nM) for 1 h at 37°C. Immediately prior to infection, MDCK cells were treated for 5 min at 37°C with the cell permeant nuclear dye Hoechst 33342 (Invitrogen) at 10 µg/mL. Cells were then infected with the virus-mAb mixture (MOI 3) mixed 1∶1 with 100 nM Lysotracker Red reagent (Molecular Probes) for 15 min at 37°C, followed by treatment with 0.05 U/well neuraminidase (Sigma Aldrich) for 5 min at 37°C. All reagents were diluted in CO_2_-independent medium supplemented with 2 mM L-glutamine. Cells were washed four times with medium before imaging live in CO_2_-independent medium supplemented with 2 mM L-glutamine and the glucose oxidase/catalase oxygen scavenging system.

### Colocalization analysis

To determine percentage colocalization between R18-labelled virus and AF647-labelled antibodies, images were analyzed using ImageJ software with the particle analysis plugin – 3D Object Counter [39].

### Overnight cell tracking

A pre-determined amount of R18-labelled H1N1 or H3N2 virus giving rise to 90–100% infection under the following experimental conditions, was pre-incubated with Alexa-Fluor 647 labeled anti-HA bnAbs to a final concentration of 30 µg/mL (200 nM) for 1 h at 37°C. MDCK cells stably expressing a GFP cell marker, were seeded into 96-well black-sided imaging plates and subsequently infected with the virus-mAb mixture for 15 min at 37°C, followed by treatment with 0.05 U/well neuraminidase for 5 min at 37°C. All reagents were diluted in CO_2_-independent medium supplemented with 2 mM L-glutamine. Cells were washed extensively before imaging live for 15 h in CO_2_-independent medium supplemented with 2 mM L-glutamine and 1% FBS. The following day, cells were fixed with ice cold 80% acetone for 10 min and stained for influenza A NP expression as previously described to confirm infection inhibition in the presence of neutralizing antibody.

### Single particle fusion assays

Fusion experiments were executed as described in the supporting information (Text S1). Briefly, R18-labeled viruses were pre-incubated with either AF488 labeled or unlabeled bnAb. A proteoliposome solution was added to the microfluidic flow cell to form a glass-supported planar lipid bilayer. Virus-bnAb mixture was added to the flow cell and viruses were immobilized onto the planar lipid bilayer (Figure S2). Fluorescein-labeled streptavidin was then added followed by washing. Viral fusion was initiated by rapid injection of a pH 5 buffer and recorded using an inverted TIRF microscope setup. Fusion events were detected as a sharp temporary increase in the fluorescence. Fusion percentage was calculated as the number of fusion events divided by the total number of virions observed in a field of view.

### HA-cleavage inhibition

To study the additive effect of HA-cleavage inhibition, uncleaved A/Wisconsin/67/05 (H3N2, MOI 3) was either first incubated with trypsin (Gibco) at 1.5 µg/mL, followed by incubation with antibodies serially diluted from 0–10 µg/mL (0–67 nM), or, first incubated with antibodies serially diluted from 0–10 µg/mL, followed by incubation with trypsin at 1.5 µg/mL. FBS was added to a final concentration of 10% after trypsin treatment to inhibit trypsin activity and all incubation steps were carried out for 45 min at 37°C. Virus-antibody mixtures were then added to confluent MDCK monolayers and allowed to incubate overnight. HA cleavage status was verified by Western blot analysis with a portion of the treated samples (data not shown). Cells were fixed with ice cold 80% acetone for 10 min and stained for influenza A NP expression as described above. Calu3 cells were infected with cleaved A/Wisconsin/67/05 (H3N2) or A/New Caledonia/20/99 (H1N1) with an MOI 3 in DMEM supplemented with 2 mM L-Glutamine. Three hours post infection cells were washed twice with PBS and incubated overnight with a concentration range (0–100 µg/mL) of test or control antibody in 50 µL medium and incubated overnight. The following day, the medium of three replicate wells was pooled and spun down for 10 min at 200× g to remove cell debris. One well from each triplicate was used to obtain cell lysate by resuspending the cell layer in 150 µL lysis buffer (50 mM Tris-HCl, 150 mM NaCl, 5 mM EDTA, 1% Triton X-100, pH 7.5). As a positive control for HA cleavage, supernatant from cells infected with virus in the absence of antibody was used and treated with 5 µg/mL trypsin for 30 min at 37°C for complete HA cleavage. Samples were then subject to Western blot analysis. To confirm viral infection, plates were also fixed and stained with ice cold 80% acetone for 10 min and stained for Influenza A NP expression.

### Statistical analyses

Single-particle fusion, and cleavage inhibition data were analyzed using a 4-parameter logistic model in which for variance stabilization the ‘*transform both sides*’ approach was used as described previously [40]. For transformation, a logit transformation was selected:
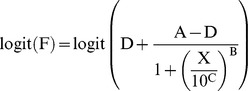
where F represents the proportion fusing virions over total virions, D and A represent respectively the upper and lower asymptote, B represents a slope factor, X represents the antibody concentration (nM) and C represents the inflection point (estimated on a log_10_ scale). For stabilization of the model fusion data obtained without antibody present was placed at an *infinite* low antibody concentration. Conditions with no events were set to 1 fusion event.

To be able to determine the effect of trypsin on the potency of CR8020, the model was modified to include an indicator variable in the estimation of the inflection point that takes the value 0 for data before trypsin and a 1 for data after trypsin (C+ID*Δc). The difference, in location, between the dose-response curves is then indicated by Δc and represents a difference in potency of the mAb under these conditions. Statistical analysis was performed using IBM SPSS statistics (version 20).

### SDS-PAGE and Immunoblotting

Relative amounts and cleavage status of hemagglutinin in the samples were determined by Western blotting. First 2 µL reducing agent (Invitrogen) and 5 µL 4× loading buffer (Ivitrogen) was added to 13 µL sample followed by 10 min incubation at 90°C. Proteins in each sample were resolved by 4–12% Bis-Tris SDS-PAGE (NUPAGE, Invitrogen) followed by trans-blotting onto a PVDF membrane (0.45 µm, P-Immobilon, Millipore, Massachusetts) in transfer buffer (NUPAGE, Invitrogen) containing 5% methanol at 30 V for 60 min. The membrane was blocked by incubation in blocking solution containing 4% non-fat dry milk (Bio-Rad) in TBST (20 mM Tris-HCl, 150 mM NaCl, 0.2% Tween 20) overnight at 4°C. The blocked membrane was incubated with rabbit anti-HA1 or -HA3 polyclonal serum, 60 ng/mL for 1 h at room temperature and washed 3 times with TBST. Subsequently, the membrane was incubated with goat anti-rabbit peroxidase conjugated F(ab′)**_2_** fragment (1∶3,000 v/v) for 1 h at room temperature. After three washes with TBST, the membranes were incubated for 5 min with ECL-Plus substrate solution (GE Healthcare). Stained proteins were visualized using Amersham Hyperfilms (GE Healthcare).

### Egress inhibition assay

Four hours prior to the experiment, 40,000 MDCK cells per well were seeded in DMEM/glutamine into flat bottom 96 well imaging plates (BD Falcon). The amount of virus needed to achieve 90–100% infection was titrated in a separate experiment. The required amount of virus was added to the cells washed twice with PBS and incubated at 37°C, 5% CO_2_. After three hours, the supernatants were removed and cells were washed twice with PBS to remove non-internalized virus particles. Cells were replenished with 50 µL infection medium containing serial diluted antibodies. After incubation for 16–18 h at 37°C, 5% CO2, the supernatants were harvested, spun down to remove debris (200× g for 10 min). The remaining cells were lysed (Tris HCl pH 7.5, 150 mM NaCl, 5 mM EDTA, 1% (v/v) Triton-X). Lysate and supernatant samples were treated with loading buffer and reducing agent, incubated for 10 min at 90°C, and analyzed by SDS-PAGE and Western blot to determine the amount of virions produced and released into the supernatant. As a control for infection, replicate identically-treated wells were fixed with 80% acetone and the number of infected cells was assessed using the imaged based infection assay (data not shown).

### Scanning electron microscopy of influenza virus infected cells

MDCK cells seeded on coverslips (sterile 15 mm thermanox plastic, Thermo Scientific, #174969) were infected with a pre-determined amount of virus (separate experiment) to yield 90–100% infected cells 18 h post infection. Three hours after the initial infection, the supernatants were removed; cells were washed thrice with PBS, before media containing the indicated concentration of antibodies were added. After an additional 15 h, the cell culture medium was removed and cells were fixed in phosphate buffered 2.5% glutaraldehyde buffer pH 7.4 for 1–2 h and stored at 4°C until further analysis. The coverslips were rinsed in PBS followed by distilled water and then dehydrated in 70%, 95%, anhydrous ethanol and finally in acetone and subjected to critical point drying in acetone and liquid CO_2_. Finally, the cells were mounted on alumina stubs and coated with a thin layer of carbon and examined in a Zeiss Ultra 55 SEM field emission microscope using an accelerating voltage of 3 keV and InLens detection at Vironova, Sweden.

### Transmission electron microscopy of influenza virus infected cells

MDCK cells seeded on coverslips were infected with a pre-determined amount of virus (separate experiment) to yield 90–100% infected cells 18 h post infection. Three hours after the initial infection, the supernatants were removed; cells were washed thrice with PBS, before media containing the indicated concentration of antibodies were added. After an additional 15 h, the cell culture medium was removed and cells were fixed in phosphate buffered 2.5% glutaraldehyde buffer for 1–2 h and stored at 4°C until further analysis. The samples were subsequently scraped and pelleted using a table top centrifuge, before being washed twice in 0.1 M phosphate buffer and chemically post-fixed with 2% osmium tetroxid (OsO4) in 0.1 M phosphate buffer for 2 h at 4°C followed by stepwise dehydration with ethanol, followed by LX 112-embedding by stepwise infiltration and polymerization at 60°C. Microtome sections of ∼60 nm were prepared and applied to one-slot formvar nickel grids. The sections were finally post-stained with uranyl acetate and Reynold's lead citrate before being imaged with a FEI Tecnai 10 electron microscope run at 100 kV accelerating voltage using a 2k x 2k Veleta CCD camera (Olympus Soft Imaging Systems) at Vironova, Sweden.

## Supporting Information

Figure S1 **Stem-binding bnAbs co-localize with influenza particles in vitro and in live cells, bind on the surface of infected cells.** (**A**) Influenza A/Puerto Rico/8/1934 (H1N1) and A/Aichi/2/1968-X31 (H3N2) viruses were labeled with the lipophilic dye octadecyl rhodamine B (R18, red), spotted onto glass, and incubated with fluorescently labeled antibodies CR6261 or CR8020. Head-binding control antibodies, CR9020 (binding to head region of a narrow spectrum of H1 HAs) and CR8057 (binding to the head region of a narrow spectrum of H3 HAs) were used in combination with R18-labeled A/New Caledonia/22/1999 (H1N1) and A/Wisconsin/67/2005 (H3N2), respectively. Antibodies CR6261 and CR8020 served as non-binding controls on H3N2 and H1N1 viruses, respectively. Virus-antibody complexes were bound to the glass bottom of 96 well plates and imaged. R18-labeled virus and AF647-labeled antibody are shown in separate channels in grayscale and in the merged image in red and green, respectively. Antibodies co-localize with the virus to which they bind *in vitro*. (**B**) Live MDCK cells expressing a GFP cell marker (grey) were incubated for ∼20 min (at 37°C) with viral particles (red) pre-incubated with antibodies and imaged as in (A). To allow detection of internalized particles only, non-internalized particles were removed by neuraminidase treatment. Whereas head-binding antibodies prevent internalization, stem-binding bnAbs co-localize with internalized viral particles (yellow). (**C**) MDCK cells were infected, fixed 15 hours later, and subsequently stained with anti-HA antibodies as in (A) and anti-influenza A nucleoprotein (NP) antibody to confirm infection (magenta, only detectable under permeabilizing conditions). Infected cells were also incubated with fluorescently labeled bnAb (green) to demonstrate their ability to bind surface-expressed HA and budding viral particles.
(TIF)

Figure S2 **CR6261 is internalized into live cells in complex with H1N1 viral particles and prevents infection).** (**A**) Separate channels (in grey scale) of a three color image showing live MDCK cells expressing a GFP-cell tracer incubated with R18-labeled A/Puerto Rico/8/1934 (H1N1) virus in complex with AF647-labeled CR6261. Internalized virus-antibody complexes (red triangles) were detected in live cells 30 min after inoculation. Individual cells were tracked over 15 hours before being fixed and stained for influenza nuclear protein (NP) to detect infection. (**B**) Control experiment showing that incubation of R18-labeled A/Puerto Rico/8/1934 (H1N1) virus with non-binding AF647-labeled CR8020 did not result in internalization of antibody. Only viral particles are detectable inside live cells 30 min after inoculation and 15 hours later these cells were infected as evident from the expression of NP.
(TIF)

Figure S3 **Calu-3 cells support the propagation of influenza virus in the absence of trypsin, but cannot be infected by uncleaved virus.** (**A**) Calu-3 cells were infected with 10 TCID_50_ cleaved A/Wisconsin/67/2005 (H3N2) influenza virus in the absence of trypsin. 24 hours after infection cells (nuclei blue) were fixed and stained for influenza NP (green) as indication for infection. (**B**) 100 TCID_50_ of uncleaved A/Wisconsin/67/2005 and A/Brisbane/59/2007 (harvested from MDCK cells in the absence of trypsin) were added to Calu-3 cells with or without trypsin. Uncleaved virus is not infectious but can be rendered infectious when treated with trypsin. Images (A and B) show an entire well.
(TIF)

Figure S4 **Influenza virus egress.** Scanning electron microscopy (SEM) and transmission electron microscopy (TEM) images of the surface of (**A**) non-infected or (**B**) influenza (B/Florida/04/2006) virus infected MDCK cells. High numbers of spherical viral particles are budding off the surface and are clearly distinguishable form microvilli or smooth cell protrusions by size, electron density, and their double membrane. Scale bar in SEM is 1 µm and in TEM 200 nm.
(TIF)

Figure S5 **HA head binding antibodies inhibit influenza virus egress.** (**A**) Calu-3 cells were infected with A/Puerto Rico/8/1934 (H1N1) and 3 hours later head-binding antibody CR9020 or 2D1 was added. Twenty hours later, the amounts of HA present in the cell supernatant (S) and lysate (L) were analyzed by Western blot (HA0 band shown). (**B**) As in (**A**) except MDCK cells were infected with B/Florida/04/2006 and the Fab fragments of CR8071 and CR8033 were used in the egress assay. (**C**) SEM images of the surface of MDCK cells infected with influenza A/California/07/2009 (H1N1), A/New Caledonia/20/1999 (H1N1), A/Wisconsin/67/2005 (H3N2), or influenza B/Florida/04/2006 virus and subsequently incubated (from 3 hours post infection) with 2D1 (5 µg/mL), CR9020 (15 µg/mL), and Zanamivir (0.5 µM) respectively. Representative images of three independent experiments are shown. Scale bar (**C**) 1 µm. (**D–E**) As in (**B**) except TEM images of ultrathin sectioned MDCK cell (re-internalized particles indicated with red triangles). Scale bar in (**D**) 500 nm and in (**E**) 100 nm.
(TIF)

Figure S6 **R18 labeled influenza virus remain infectious.** MDCK cells were infected with R18- or MOCK-labeled A/Puerto Rico/8/1934 (H1N1) or A/Aichi/2/1968-X31 (H3N2) and the number of infected cells (nucleus stained with DAPI, blue) for each virus was determined by staining for influenza NP expression (green).
(TIF)

Movie S1 **Stem-binding bnAb CR8020 is internalized into live cells in complex with H3N2 virus particles.** R18-labeled A/Aichi/2/68-X31 (H3N2) virus particles pre-incubated with AF647-labeled CR8020 are internalized into live MDCK cells (nucleus, blue). Movie (∼27 min past incubation, mpi) shows the directed motion of virus particles (red) together with mAbs (green) along *TubulinTracker*-stained microtubules (white).
(AVI)

Movie S2 **Stem-binding bnAb CR6261 is internalized into live cells in complex with H1N1 virus particles.** R18-labeled A/Puerto Rico/8/34 (H1N1) virus particles pre-incubated with AF647-labeled CR6261 are internalized into live MDCK cells (nucleus, blue). Movie (∼40 mpi) shows the directed motion of virus particles (red) together with mAbs (green) along *TubulinTracker*-stained microtubules (white).
(AVI)

Movie S3 **Stem-binding bnAb CR8020 is not internalized after incubation with H1N1 virus particles.** After incubation of R18-labeled A/Puerto Rico/8/34 (H1N1) virus particles (red) with AF647-labeled non-binding control antibody CR8020, only virus particles are internalized ∼33 mpi into live MDCK cells (nucleus, blue).
(AVI)

Movie S4 **Stem-binding bnAb CR6261 is not internalized after incubation with H3N2 virus particles.** After incubation of R18-labeled A/Aichi/2/68-X31 (H3N2) virus particles (red) with AF647-labeled non-binding control antibody CR6261, only virus particles are internalized ∼41 mpi into live MDCK cells (nucleus, blue).
(AVI)

Movie S5 **H1N1 virus incubated with only 15 nM CR6261-AF488 can undergo fusion.** Representative portions of dual-color fluorescence viral fusion recordings obtained with 200 ms exposure times; scale bar equals 2 µm. R18-labeled A/Puerto Rico/8/34 (H1N1) virus (false colored magenta, center column) incubated for 30 min with 15 nM AF488-labeled CR6261 (green, right column). Co-localization between the virus and bound bnAb (white) is shown in the left column (merge). Time *t* = 0 indicates drop of pH from 7.4 to 5.0. Fusion events are observed as the rapid increase in fluorescence signal (dequenching) at the site of a virus, followed by quick, outward diffusion of the lipophilic R18 dye away from the fusion site. Both movies (S5 and S6) were recorded under identical illumination conditions. Contrast settings of the 15 nM bnAb incubation has been enhanced 25% relative to the 1500 nM incubation (Movie S6). All images were scaled 4-fold larger using bicubic interpolation. For assay details see experimental procedures.
(AVI)

Movie S6 **H1N1 virus incubated with 1500 nM CR6261-AF488 is fusion incompetent.** As in (S5) except, R18-A/Puerto Rico/8/34 (H1N1, magenta) was incubated with 1500 nM AF488-labeled CR6261 (green). The higher bnAb concentration inhibited HA-mediated fusion and no dequenching or R18 diffusion is observed. Both movies (S5 and S6) were recorded under identical illumination conditions.
(AVI)

Table S1 **Characteristics of broadly neutralizing antibodies, control antibodies, and Fab fragments used in this study.**
(DOC)

Table S2 **Colocalization of virus-antibody complexes in infected MDCK cells.**
(DOC)

Text S1 **Supporting Materials and Methods for Single particle fusion assays.**
(DOC)
