# Supplementary figures and images for: Mechanisms of Hemagglutinin Targeted Influenza Virus Neutralization
Source: PLoS One. 2013 Dec 11;8(12):e80034. doi: 10.1371/journal.pone.0080034 (PMC3862845; doi:10.1371/journal.pone.0080034)

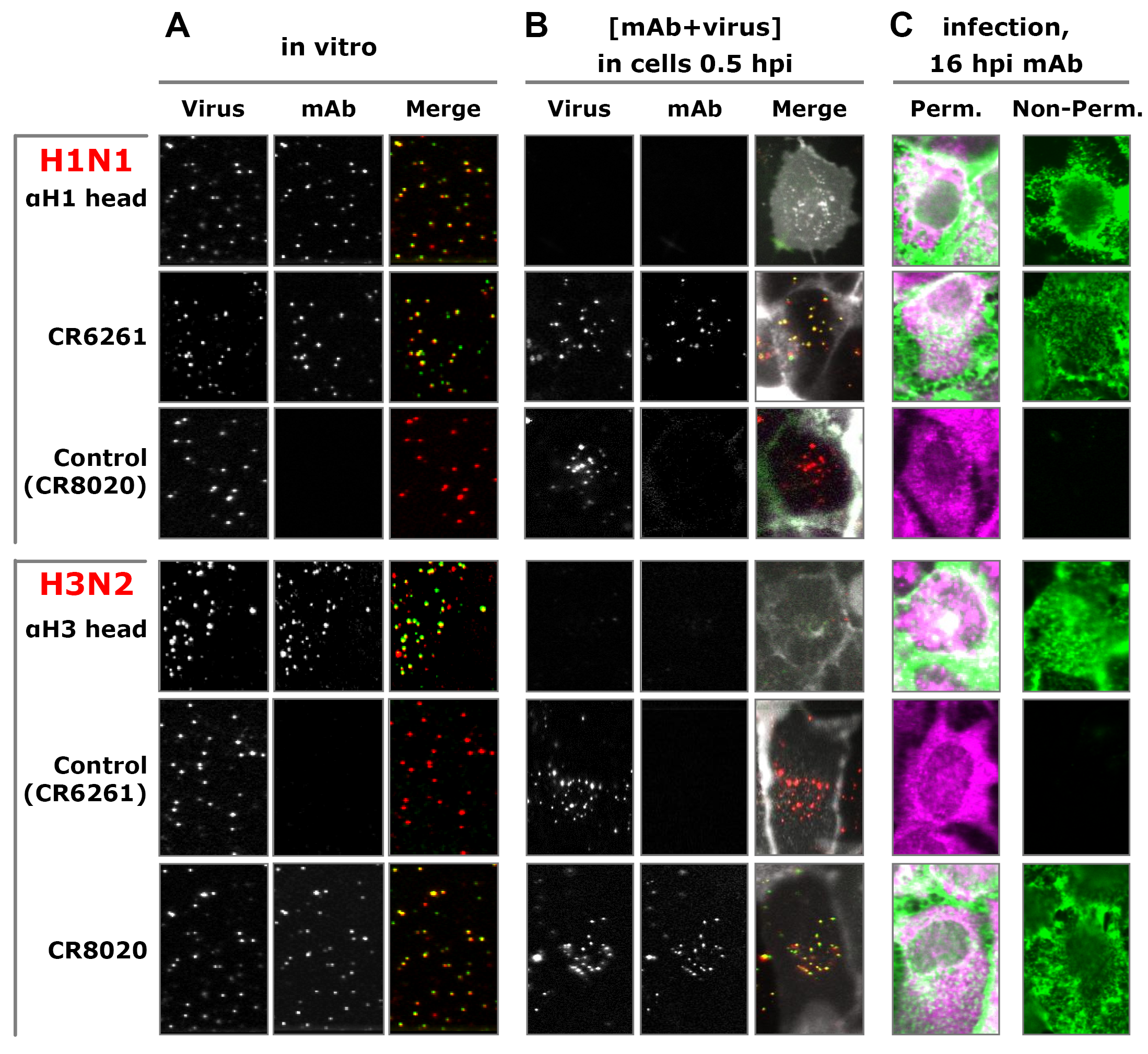

Supplement: Figure S1 — Stem-binding bnAbs co-localize with influenza particles in vitro and in live cells, bind on the surface of infected cells. (A) Influenza A/Puerto Rico/8/1934 (H1N1) and A/Aichi/2/1968-X31 (H3N2) viruses were labeled with the lipophilic dye octadecyl rhodamine B (R18, red), spotted onto glass, and incubated with fluorescently labeled antibodies CR6261 or CR8020. Head-binding control antibodies, CR9020 (binding to head region of a narrow spectrum of H1 HAs) and CR8057 (binding to the head region of a narrow spectrum of H3 HAs) were used in combination with R18-labeled A/New Caledonia/22/1999 (H1N1) and A/Wisconsin/67/2005 (H3N2), respectively. Antibodies CR6261 and CR8020 served as non-binding controls on H3N2 and H1N1 viruses, respectively. Virus-antibody complexes were bound to the glass bottom of 96 well plates and imaged. R18-labeled virus and AF647-labeled antibody are shown in separate channels in grayscale and in the merged image in red and green, respectively. Antibodies co-localize with the virus to which they bind in vitro. (B) Live MDCK cells expressing a GFP cell marker (grey) were incubated for ∼20 min (at 37°C) with viral particles (red) pre-incubated with antibodies and imaged as in (A). To allow detection of internalized particles only, non-internalized particles were removed by neuraminidase treatment. Whereas head-binding antibodies prevent internalization, stem-binding bnAbs co-localize with internalized viral particles (yellow). (C) MDCK cells were infected, fixed 15 hours later, and subsequently stained with anti-HA antibodies as in (A) and anti-influenza A nucleoprotein (NP) antibody to confirm infection (magenta, only detectable under permeabilizing conditions). Infected cells were also incubated with fluorescently labeled bnAb (green) to demonstrate their ability to bind surface-expressed HA and budding viral particles. (TIF) [file pone.0080034.s001.tif]

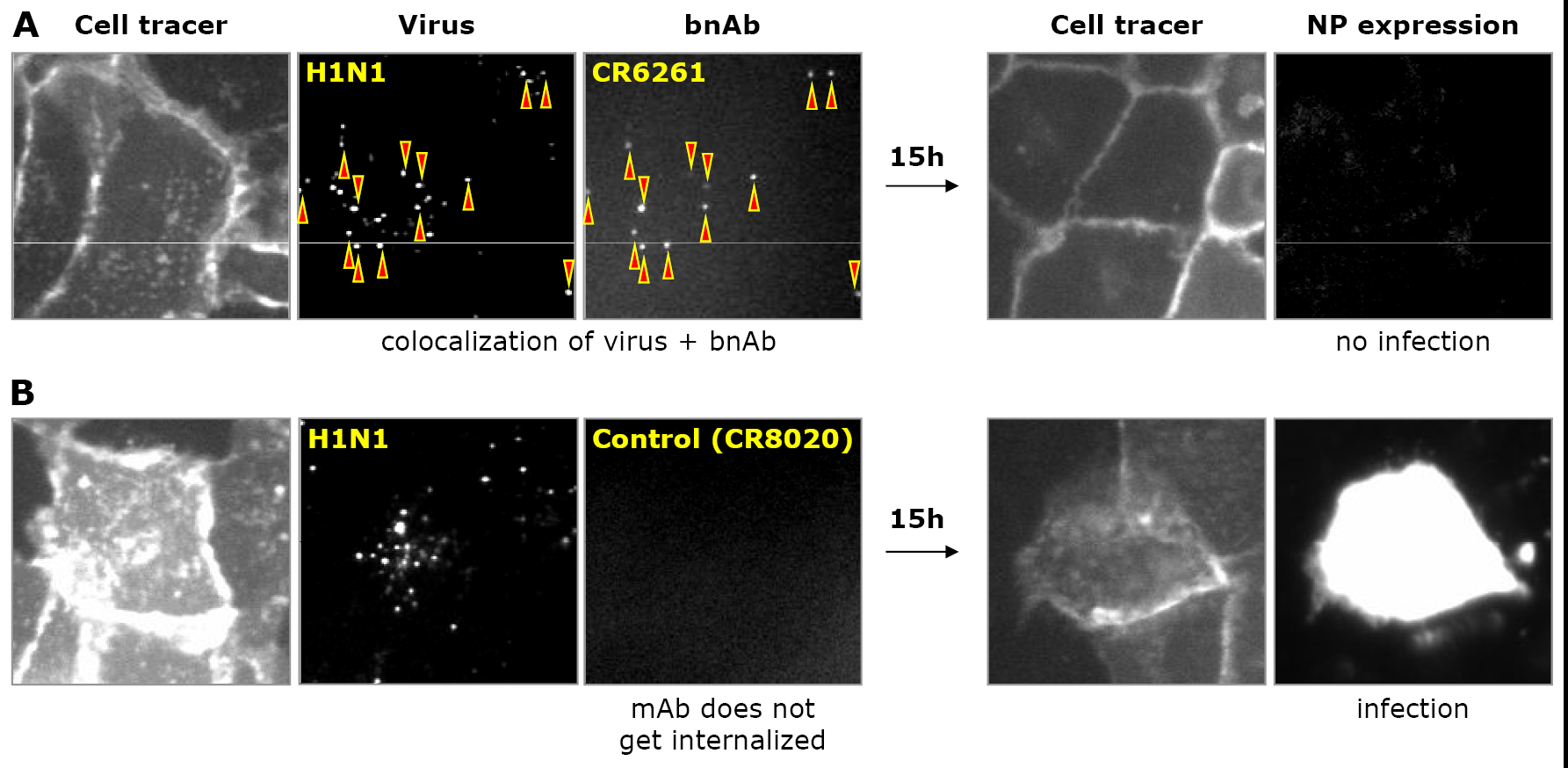

Supplement: Figure S2 — CR6261 is internalized into live cells in complex with H1N1 viral particles and prevents infection). (A) Separate channels (in grey scale) of a three color image showing live MDCK cells expressing a GFP-cell tracer incubated with R18-labeled A/Puerto Rico/8/1934 (H1N1) virus in complex with AF647-labeled CR6261. Internalized virus-antibody complexes (red triangles) were detected in live cells 30 min after inoculation. Individual cells were tracked over 15 hours before being fixed and stained for influenza nuclear protein (NP) to detect infection. (B) Control experiment showing that incubation of R18-labeled A/Puerto Rico/8/1934 (H1N1) virus with non-binding AF647-labeled CR8020 did not result in internalization of antibody. Only viral particles are detectable inside live cells 30 min after inoculation and 15 hours later these cells were infected as evident from the expression of NP. (TIF) [file pone.0080034.s002.tif]

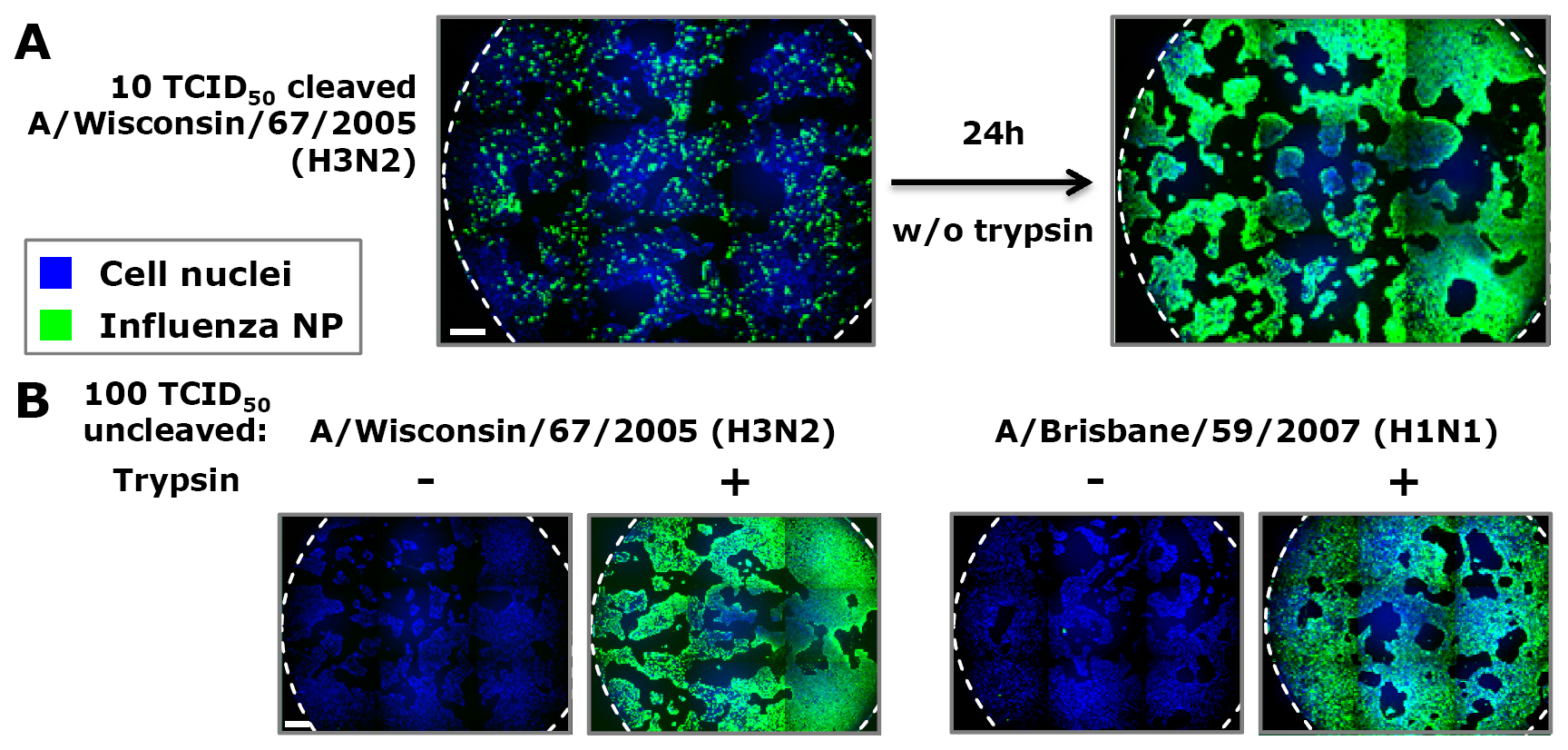

Supplement: Figure S3 — Calu-3 cells support the propagation of influenza virus in the absence of trypsin, but cannot be infected by uncleaved virus. (A) Calu-3 cells were infected with 10 TCID50 cleaved A/Wisconsin/67/2005 (H3N2) influenza virus in the absence of trypsin. 24 hours after infection cells (nuclei blue) were fixed and stained for influenza NP (green) as indication for infection. (B) 100 TCID50 of uncleaved A/Wisconsin/67/2005 and A/Brisbane/59/2007 (harvested from MDCK cells in the absence of trypsin) were added to Calu-3 cells with or without trypsin. Uncleaved virus is not infectious but can be rendered infectious when treated with trypsin. Images (A and B) show an entire well. (TIF) [file pone.0080034.s003.tif]

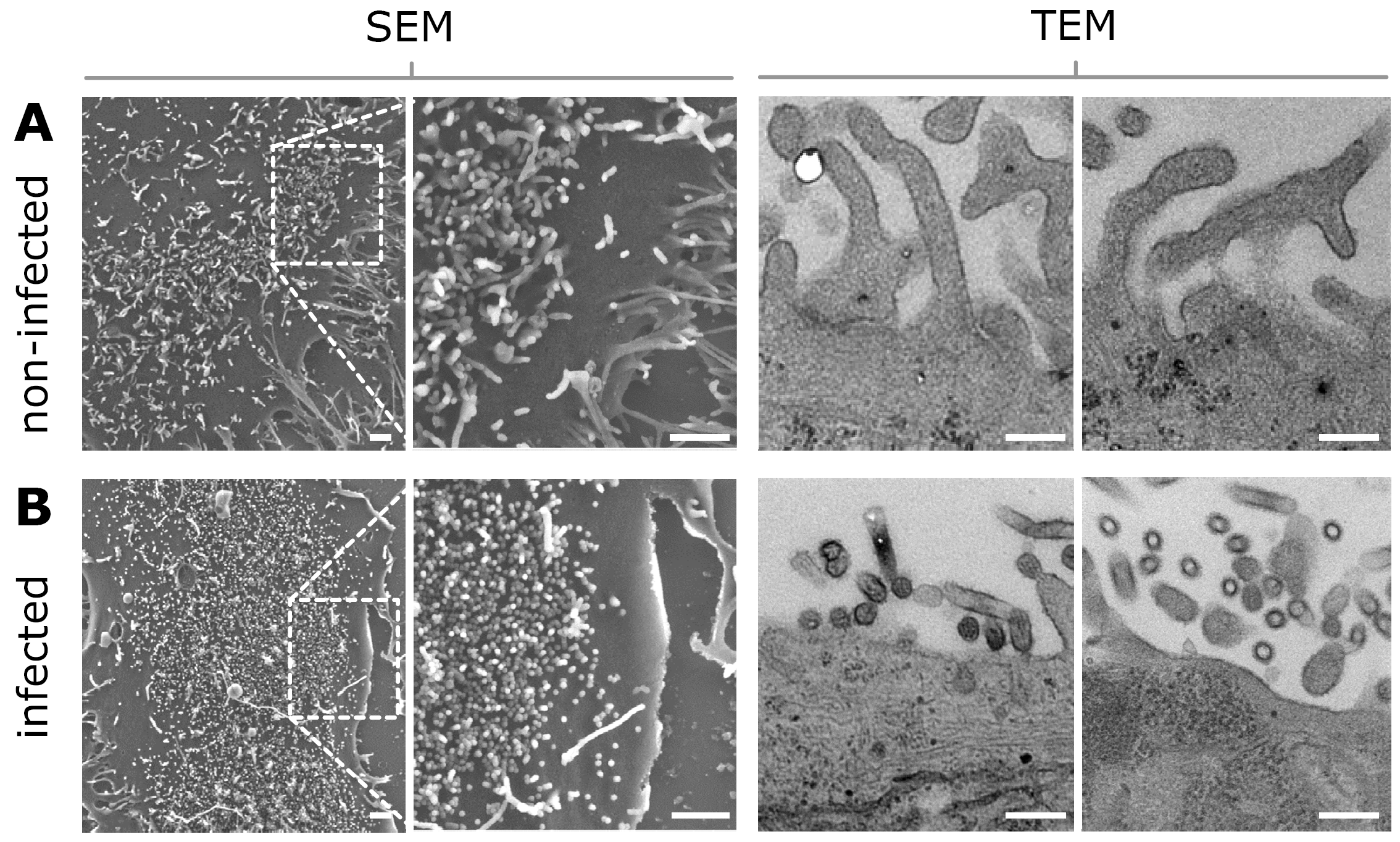

Supplement: Figure S4 — Influenza virus egress. Scanning electron microscopy (SEM) and transmission electron microscopy (TEM) images of the surface of (A) non-infected or (B) influenza (B/Florida/04/2006) virus infected MDCK cells. High numbers of spherical viral particles are budding off the surface and are clearly distinguishable form microvilli or smooth cell protrusions by size, electron density, and their double membrane. Scale bar in SEM is 1 µm and in TEM 200 nm. (TIF) [file pone.0080034.s004.tif]

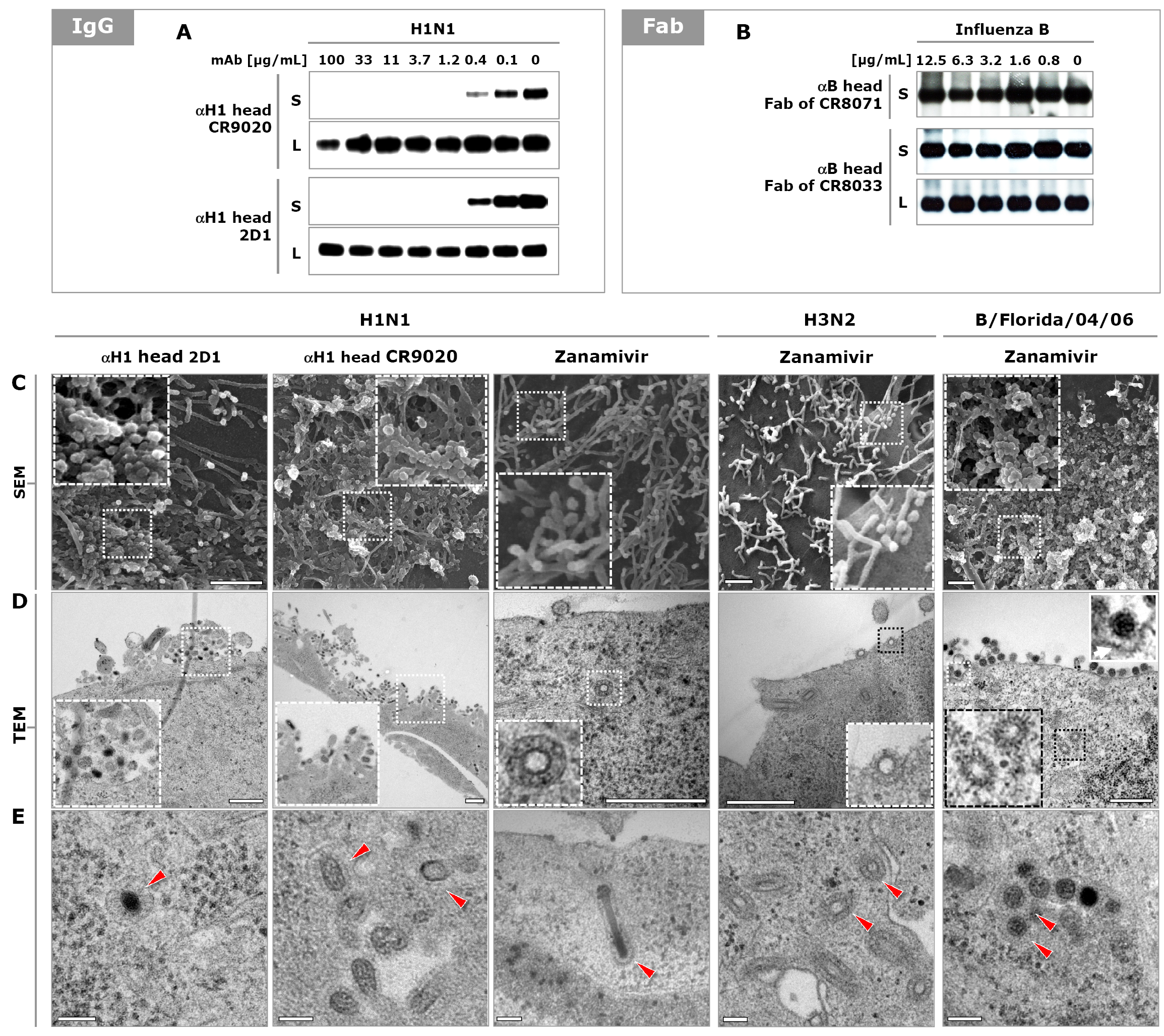

Supplement: Figure S5 — HA head binding antibodies inhibit influenza virus egress. (A) Calu-3 cells were infected with A/Puerto Rico/8/1934 (H1N1) and 3 hours later head-binding antibody CR9020 or 2D1 was added. Twenty hours later, the amounts of HA present in the cell supernatant (S) and lysate (L) were analyzed by Western blot (HA0 band shown). (B) As in (A) except MDCK cells were infected with B/Florida/04/2006 and the Fab fragments of CR8071 and CR8033 were used in the egress assay. (C) SEM images of the surface of MDCK cells infected with influenza A/California/07/2009 (H1N1), A/New Caledonia/20/1999 (H1N1), A/Wisconsin/67/2005 (H3N2), or influenza B/Florida/04/2006 virus and subsequently incubated (from 3 hours post infection) with 2D1 (5 µg/mL), CR9020 (15 µg/mL), and Zanamivir (0.5 µM) respectively. Representative images of three independent experiments are shown. Scale bar (C) 1 µm. (D–E) As in (B) except TEM images of ultrathin sectioned MDCK cell (re-internalized particles indicated with red triangles). Scale bar in (D) 500 nm and in (E) 100 nm. (TIF) [file pone.0080034.s005.tif]

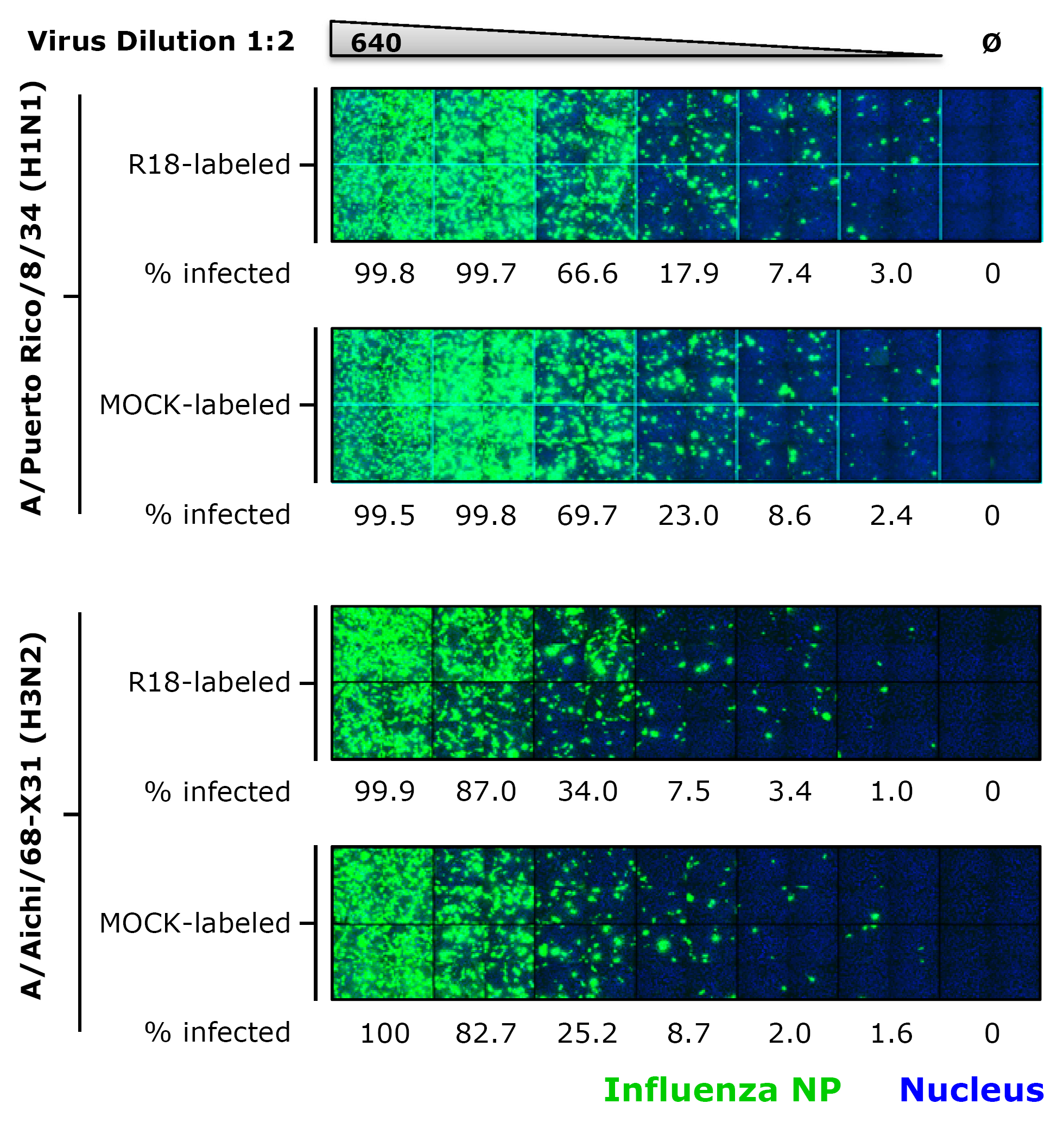

Supplement: Figure S6 — R18 labeled influenza virus remain infectious. MDCK cells were infected with R18- or MOCK-labeled A/Puerto Rico/8/1934 (H1N1) or A/Aichi/2/1968-X31 (H3N2) and the number of infected cells (nucleus stained with DAPI, blue) for each virus was determined by staining for influenza NP expression (green). (TIF) [file pone.0080034.s006.tif]
