# Supplementary material for: Mechanisms of Hemagglutinin Targeted Influenza Virus Neutralization
Source: PLoS One. 2013 Dec 11;8(12):e80034. doi: 10.1371/journal.pone.0080034 (PMC3862845; doi:10.1371/journal.pone.0080034)
Supplement: Table S1 — Characteristics of broadly neutralizing antibodies, control antibodies, and Fab fragments used in this study. (DOC) [file pone.0080034.s013.doc]

**Table S1. Characteristics of bnAbs, control antibodies, and Fab fragments used in this study.**

|  |  | **VNA titer [µg/mL]** | | | **HAI titer [µg/mL]** | | |  |  |
| --- | --- | --- | --- | --- | --- | --- | --- | --- | --- |
| **IgG1/ Fab** | **HA Epitope** | **H1N1** | **H3N2** | **B** | **H1N1** | **H3N2** | **B** | **Breadth^[h]^** | **Ref.** |
| CR6261 | Stem | 3.58 ^[a]^ | - | - | - | - | - | broad A-G1 | [5] |
| CR8020 | Stem | - | 2.2 ^[b]^ | - | - | - | - | broad A-G2 | [6] |
| CR8033 | Head | - | - | 0.02 ^[c]^ | - | - | 0.22 ^[c]^ | broad B | [7] |
| CR8033 Fab | Head | - | - | 2.63 ^[c]^ | - | - | 4.42 ^[c]^ | broad B | [7] |
| CR8071 | Head | - | - | 2.04 ^[c]^ | - | - | - | broad B | [7] |
| CR8071 Fab | Head | - | - | - ^[k]^ | - | - | - | broad B | [7] |
| CH65^[g]^ | Head | 0.44 ^[d]^ | - | - | 0.88 ^[d]^ | - | - | broad H1 | [8] |
| CH65 Fab | Head | 4.20 ^[d]^ | - | - | 17.7 ^[d]^ | - | - | broad H1 | [8] |
| 2D1^[g]^ | Head | 0.08 ^[e]^ | - | - | 0.22 ^[e]^ | - | - | narrow H1 | [9] |
| CR9020 | Head | <0.01 ^[d]^ | - | - | 0.22 ^[d]^ | - | - | narrow H1 | [i] |
| CR8057 | Head | - | <0.01 ^[f]^ | - | - | 0.03 ^[f]^ | - | narrow H3 | [6] |
| CR8057 Fab | Head | - | <0.02 ^[f]^ | - | - | 0.03 ^[f]^ | - | narrow H3 | [6] |

[a] A/Puerto Rico/8/34

[b] A/Aichi/2/68-X31

[c] B/Florida/04/06

[d] A/New Caledonia/20/99

[e] NYMC X-181 (6:2 reassortant of PR8 with the HA and NA segments of A/California/07/09)

[f] A/Wisconsin/67/2005

[g] The variable heavy and light chains of CH65 and 2D1 were cloned into Crucell’s human IgG backbone. The resulting IgGs (CR11055 and CR11054, respectively) have identical specificity as the published antibodies.

[h] A = Influenza A virus, G1 = influenza A virus group 1 (comprises the H1, H2, H5, H6, H8, H9, H11, H12, H13, H16 and H17 subtypes), G2 = influenza A virus group 2 (comprises the H3, H4, H7, H10, H14 and H15 subtypes), B = influenza B virus

[i] This study

^[k]^  CR8071 Fab efficiently binds to influenza B HA [7]

– No activity
