# Supplementary material for: Mechanisms of Hemagglutinin Targeted Influenza Virus Neutralization
Source: PLoS One. 2013 Dec 11;8(12):e80034. doi: 10.1371/journal.pone.0080034 (PMC3862845; doi:10.1371/journal.pone.0080034)
Supplement: Table S2 — Colocalization of virus-antibody complexes in infected MDCK cells. (DOC) [file pone.0080034.s014.doc]

**Table S2. Colocalization of virus-antibody complexes in infected MDCK cells**

| **Virus** | **Strain** | **bnAb** | **# cells counted** | **# particles counted** | **% colocalization virus + bnAb** | **StDev** |
| --- | --- | --- | --- | --- | --- | --- |
| H1N1 | A/PR/8/1934 | CR6261 | 175 | 605 | 93.5 | 1.9 |
| H1N1 | A/PR/8/1934 | CR8020 | 142 | 582 | 0.2 | 0.5 |
| H3N2 | A/Aichi/1968-X31 | CR8020 | 159 | 1257 | 92.0 | 5.6 |
| H3N2 | A/Aichi/1968-X31 | CR6261 | 153 | 1158 | 0.14 | 0.3 |
