# Supplementary material for: Mechanisms of Hemagglutinin Targeted Influenza Virus Neutralization
Source: PLoS One. 2013 Dec 11;8(12):e80034. doi: 10.1371/journal.pone.0080034 (PMC3862845; doi:10.1371/journal.pone.0080034)
Supplement: Text S1 — Supporting Materials and Methods for Single particle fusion assays. (DOC) [file pone.0080034.s015.doc]

**Mechanisms of hemagglutinin targeted influenza virus neutralization**

Supporting Information

Boerries Brandenburg^1^, Wouter Koudstaal^1^, Jaap Goudsmit^1^, Vincent Klaren^1^, Chan Tang^1^, Miriam V. Bujny^1^, Hans J. W. M. Korse^1^, Ted Kwaks^1^, Jason J. Otterstrom^2,3^, Jarek Juraszek^1^, Antoine M. van Oijen^2^, Ronald Vogels^1^ and Robert H. E. Friesen^1*^

^1^ Crucell Vaccine Institute, Janssen Center of Excellence for Immunoprophylaxis, Leiden, The Netherlands

^2^ Centre for Synthetic Biology, Zernike Institute for Advanced Materials, Groningen, The Netherlands

^3^ Harvard Biophysics Program, Harvard Medical School, Boston, MA, USA

^*^ Correspondence to: Robert H. E. Friesen, Archimedesweg 4-6; 2333 CN Leiden, The Netherlands. Telephone +31715197464, Fax +31715199102

E-mail: RFriesen@its.jnj.com

**Supporting Materials and Methods**

**Single particle fusion assays.**

***Microfluidic flow cell construction.*** Microfluidic flow cells were constructed for fusion experiments using cleaned glass microscope cover slips (25 x 25-mm, No. 1, VWR) and either a PDMS (polydimethylsiloxane; Dow Corning, Slygard 184) chip or a quartz top affixed with double sided tape (Grace Bio-Labs) as described previously [1, 2]. A PDMS chip was used with unlabeled antibodies, while the quartz was used for AF488-labeled antibody experiments. PDMS chips formed by standard PDMS cast molding techniques [3] housed five parallel channels of dimensions 0.5(w) x 0.2 (h) x 10(l) mm and were non-permanently adsorbed to the clean glass cover slips. Holes were punched into the chip to allow insertion the PE20 inlet and PE60 outlet tubing (BD Intramedic; I.D = 0.38 mm and 0.76 mm, respectively). The PDMS chip with glass bottom was clamped in a home-built PDMS-chip housing and affixed to the microscope stage with a custom adaptor.

***Proteoliposome Preparation.*** Chloroform solutions of 1,2-dioleoyl-sn-glycero-3-phosphocholine (DOPC) (Avanti Polar Lipids), cholesterol (Avanti Polar Lipids) and *N*-((6-(biotinoyl)amino)hexanoyl)-1,2-dihexadecanoyl-*sn*-glycero-3-phosphoethanolamine triethylammonium salt (Biotin-X DHPE, Invitrogen) were mixed in a molar ratio of 0.8:0.2:2.5x10^-5^. The lipid mixture was dried under an argon stream, then for two hours under vacuum. Lipids were resuspended in HNE buffer to 5 mg/mL, freeze/thawed five times with liquid nitrogen, then extruded (mini-extruder, Avanti) using 0.2 µm pore size filters at ~40 °C. Triton X-100 (VWR) was added to a final concentration of 0.5 % (w/v) and incubated in the liposome suspension at 37 °C for 15 min then on ice for 15 min. Sialoglycoprotein glycophorin A (GYPA) – full-length recombinant protein with a GST tag (Abnova) – was added to solubilized lipids at a lipid:protein molar-ratio of approximately 1:40,000 and allowed to mix at 4 °C for 30 min. Triton-X detergent was removed by two successive two hour incubations with Bio-Beads (SM-2 absorbent, Bio-Rad Laboratories, Inc.) at 4 °C using 200 mg Bio-Beads per 300 µL of solution. Glycophorin A membrane protein was used in lieu of membrane-bound gangliosides, such as GD1a, because we found that the H1N1 virus strain did not become immobilized upon planar bilayers containing sialic acid presented in this fashion.

***Microscope Setup.*** Single-particle fusion assays were conducted on an inverted dual color fluorescent microscope (Olympus IX-71) equipped with a 60x NA 1.49 oil immersion objective (Olympus APON60OTIRF), and both 488 nm blue and 561 nm yellow continuous-wave solid state lasers (Sapphire models, Coherent Inc.) aligned in objective-based Hi-Lo total internal reflection (TIR) mode [4]. Emitted fluorescence light was filtered using a custom-ordered microscope filter cube (Chroma Ltd.) allowing passage of wavelengths: 495 to 550 and 570 to 710. The green fluorescein/AF488 and red R18 emitted fluorescence signals were spectrally separated in a home-build dual view setup utilizing a long-pass dichroic mirror (Thorlabs DMLP567), passed through band-pass filters (Chroma ET525-50m and ET605-70m, respectively) and focused onto either half of an EMCCD camera (Hamamatsu Photonics K.K., Image-EM model C9100-13).

***Fusion Experiment.*** R18-labeled viruses were diluted 10-fold into a solution of either 100 % Alexa Fluor-488 labeled or 100 % unlabeled antibodies, bringing the antibodies to the desired final concentration, and then incubated at room temperature for 30 min prior to flow cell addition. The proteoliposome solution was added to the microfluidic flow cell mounted to the microscope stage using negative pressure from a syringe pump (New Era Pump Systems Inc., NE-1000) connected via a six-valve manifold (Qosina, Edgewood, NY, USA) and allowed to incubate for 30 min at room temperature, spontaneously forming a glass-supported planar lipid bilayer Figure 2A). The fusion experiments were executed as reported previously [1, 2] in a fashion similar to wherein virus in the antibody solution was added to the flow cell and viruses were observed to immobilize upon the fluid, continuous, planar bilayer. Fluorescein-labeled streptavidin (Invitrogen) was subsequently added at a concentration of 6 µg/mL with unlabeled antibodies or at 0.2 µg/mL with labeled. This was followed by a 2 minute wash at 100 µL/min with clean HNE buffer to remove unbound viruses, unbound antibodies and unbound streptavidin-fluorescein. Viral fusion was initiated by rapid injection of a citric acid buffer (10 mM citric acid, 140 mM NaCl, 0.2 mM EDTA, pH 5.0) at 200 µL/min and recorded using Metavue imaging software (Life Science Imaging Ltd.) at an acquisition rate of 5 Hz and maximal EM gain until all fusion events had ceased. Images in figure 2 and supplementary movies S5 and S6 were created using ImageJ.

***Data Analysis.*** Recorded fusion movies were processed and analyzed in a fashion similar to that previously described [1, 2]. Individual viral particles were identified and their fluorescent trajectories extracted using home-written MATLAB code. Arrival of the acidic buffer led to disappearance of the fluorescein signal and synchronization of viral fusion. The onset of fusion between the virus and target bilayer was visualized by sudden increases in the R18 signal, caused by fluorescence dequenching, followed by outward diffusion of the R18 molecules into the target bilayer away from the fusion site. Red-channel fluorescent trajectories (fluorescence at a spot over time) of the viruses were extracted from recorded movies and plotted for manual selection. Trajectories showing clear dequenching spikes followed by dissipative signal loss were directly classified as fusing virions. Trajectories showing characteristics of dequenching and/or dissipative signal loss, but with poorer signal strength, were subjected to further manual inspection. In this case, a virus particle in question is observed in the recorded fusion movie. Viruses showing a rapid outward movement of lipid molecules away from the virus identifiable by eye were also classified as fusing virions. The percent hemifusion in each experiment was calculated as the number of particles in a field of view determined to undergo fusion divided by the total number of particles initially detected in the same field of view. Experiments of fusion at each antibody concentration were conducted at least three times and experiments having fewer than 25 particles detected in a field of view were not included in the final analysis. Additionally, experiments showing 25 fusing particles or fewer were subjected to two rounds of particle selection to reduce the likelihood of false-event selection.

**References**

1. Floyd DL, Ragains JR, Skehel JJ, Harrison SC, van Oijen AM (2008) Single-particle kinetics of influenza virus membrane fusion. Proc Natl Acad Sci U S A 105: 15382-15387.

2. Floyd DL, Harrison SC, van Oijen AM (2009) Method for measurement of viral fusion kinetics at the single particle level. J Vis Exp 31: e1484.

3. Qin D, Xia Y, Whitesides GM (2010) Soft lithography for micro- and nanoscale patterning. Nat Protoc 5: 491-502.

4. Tokunaga M, Imamoto N, Sakata-Sogawa K (2008) Highly inclined thin illumination enables clear single-molecule imaging in cells. Nat Methods 5: 159-161.

5. Throsby M, van den Brink E, Jongeneelen M, Poon LL, Alard P, et al. (2008) Heterosubtypic neutralizing monoclonal antibodies cross-protective against H5N1 and H1N1 recovered from human IgM+ memory B cells. PLoS One 3: e3942.

6. Ekiert DC, Friesen RH, Bhabha G, Kwaks T, Jongeneelen M, et al. (2011) A highly conserved neutralizing epitope on group 2 influenza A viruses. Science 333: 843-850.

7. Dreyfus C, Laursen NS, Kwaks T, Zuijdgeest D, Khayat R, et al. (2012) Highly conserved protective epitopes on influenza B viruses. Science 337: 1343-1348.

8. Whittle JR, Zhang R, Khurana S, King LR, Manischewitz J, et al. (2011) Broadly neutralizing human antibody that recognizes the receptor-binding pocket of influenza virus hemagglutinin. Proc Natl Acad Sci U S A 108: 14216-14221.

9. Yu X, Tsibane T, McGraw PA, House FS, Keefer CJ, et al. (2008) Neutralizing antibodies derived from the B cells of 1918 influenza pandemic survivors. Nature 455: 532-536.
